# Yeast-based attract-and-kill strategies for *Drosophila suzukii* management without disrupting honey bee activity

**DOI:** 10.1371/journal.pone.0323653

**Published:** 2025-05-19

**Authors:** Claire Duménil, Urban Spitaler, Guillermo Rehermann, Flavia Bianchi, Riccardo Favaro, Irene Castellan, Silvia Schmidt, Daniela Eisenstecken, Paul G. Becher, Sergio Angeli

**Affiliations:** 1 Faculty of Agricultural, Environmental and Food Sciences, Free University of Bozen-Bolzano, Piazza Università 1, Bolzano, Italy; 2 Institute for Plant Health, Laimburg Research Centre, Laimburg 6, Auer-Ora, Italy; 3 Department of Plant Protection Biology, Chemical Ecology Horticulture Unit, Swedish University of Agricultural Sciences, Alnarp, Sweden; 4 Laboratory for Flavours and Metabolites, Institute for Agricultural Chemistry and Food Quality, Laimburg Research Centre, Laimburg 6, Auer-Ora, Italy; University of Thessaly School of Agricultural Sciences, GREECE

## Abstract

Attract-and-kill strategies are effective, sustainable pest control methods. Formulations combining the insecticide spinosad, at a lower dose than conventional methods, with the *Drosophila*-associated yeast *Hanseniaspora uvarum* have shown promising results*.* Recently, *Saccharomycopsis vini* was identified as the most attractive yeast for ovipositing females. In this study, the potential of *S. vini* for use in attract-and-kill formulations against *D. suzukii* was evaluated alongside *H. uvarum*. Behavioural assays demonstrated that *D. suzukii* preferred *S. vini* when both yeasts are simultaneously present in a close range setting but was attracted to both in long range attraction assays. In efficacy assays, *S. vini* and *H. uvarum* were equally efficient at reducing oviposition and increasing mortality in formulation with spinosad. Offering yeast formulations at the foraging sites of trained honey bees did not stimulate more feeding when compared to sugar syrup. The characterisation of the organic volatile compounds released from the cultures demonstrated that *S. vini* and *H. uvarum* were composed of overlapping as well as distinct chemicals. The antennally active compounds ethyl acetate and ethyl propanoate were abundant in the more attractive *S. vini* and *H. uvarum*, while the compounds 3-methyl-1-butanol and 2-methylthiolan-3-one were more abundant in the less attractive *S. cerevisiae*. These chemicals may be further studied as possible attractants or repellents for *D. suzukii.* We propose *S. vini* as a new yeast with potential for use in integrated pest management, with a distinctive volatile profile while maintaining a similar efficacy compared to *H. uvarum* against *D. suzukii.* Neither *H. uvarum* nor *S. vini* stimulated honey bee foraging behaviour, suggesting that both yeast-based attract-and-kill formulations pose a low non-target risk to honey bees.

## Introduction

The spotted wing drosophila, *Drosophila suzukii* Matsumura (Diptera: Drosophilidae) is an invasive insect pest that lays eggs and develops in the ripening fruits of more than 50 different plant species [1,2]. Females puncture the skin of the fruit to lay eggs in the flesh, where larvae begin feeding. In infested fruits, *D. suzukii* interacts with microbial communities, including yeast species that act as feeding and oviposition stimulants [3,4] and play a crucial role in the biology of this insect [5]. Flies rely on volatile organic compounds (VOCs) released from host fruits and associated microbes to detect suitable habitats using a highly specialised olfactory system [6–8].

Due to its ability to attack soft-skinned fruits, *D. suzukii* causes significant economic losses, rendering fruits unmarketable [9,10]. Organic and integrated agriculture practices are being developed to complement or replace synthetic insecticides. The bacterial-derived spinosad has demonstrated high efficacy against *D. suzukii* on several crops and is approved for use in organic agriculture [2,11]. However, applying spinosad during the late ripening stage raises concerns about undesirable pesticide residues on commercialised fruits [12]. It has already led to increased resistance in *D. suzukii* due to its intensive use [13], and its potential harm to non-target species [14,15].

To mitigate these issues while maintaining high efficacy, attract-and-kill strategies are being developed. These strategies combine lower amounts of applied insecticides with alternative methods, such as the addition of natural VOCs that trigger attractive behaviours, and lure insects with high specificity into localised lethal traps [16–19]. Such strategies have shown positive results, using fruit- and *Drosophila*-associated yeasts as attractive sources. These yeasts not only attract flies but also stimulate oviposition, as noted with the yeasts *Hanseniaspora uvarum* and *Saccharomyces cerevisiae* [20–22] making them effective in luring target species to a lethal source.

The yeast *H. uvarum,* used in synergy with spinosad in vineyards, has proved to attract and kill more flies while using a lower dose of insecticide compared to conventional methods. It has also been more efficient than any other yeast tested to date [23–27]. Another promising yeast species is *Saccharomycopsis vini*, isolated from *D. suzukii-*infested fruits [20]. This yeast has been shown to stimulate feeding and fecundity in *D. suzukii* females [28], and when compared with *H. uvarum* and other yeasts, it was found to be the most attractive in laboratory assays [29].

In this study, we assessed the potential of *S. vini* to be used in attract-and-kill formulations against *D. suzukii*. We conducted a comparative study to evaluate the attractiveness of both yeast cultures and their efficacy when formulated with spinosad. In addition, the attractiveness of both yeasts to honey bees was tested for the first time. A comparative analysis of headspace volatiles of *H. uvarum, S. vini* and the less attractive yeast, *S. cerevisiae,* was performed to understand how flies discriminate between yeast cultures and to identify which headspace compounds are detected by *D. suzukii*. The results could contribute to the development of novel tools to diversify or improve pest management programmes.

## Materials and methods

### *Drosophila suzukii* rearing

*Drosophila suzukii* were reared in insect cages (W47.5 × D47.5 × H93.0 cm, BugDorm – 4 M4590, MegaView Science Co., Ltd., Taichung, Taiwan) on a cornmeal diet (DSCD(a) containing dry deactivated yeast), supplemented with dry baker’s yeast (RUF Lebensmittelwerk KG, Quakenbrück) and an additional 5% sugar solution provided on cotton, under a 16:8 h L:D photoperiod [20]. For all experiments, 4–8-day-old adults were used. The *D. suzukii* colony was established from field-infested cherries, blueberries, and grapes collected in South Tyrol, Italy, and was refreshed annually with field-collected individuals.

### Apis mellifera rearing

*Apis mellifera* subsp. *carnica* (Pollmann) were kept in the experimental apiary of the Free University of Bolzano, located in Altenburg (46°23′12.6″N 11°13′57.5″E, South Tyrol, Italy). Five colonies of similar strength were created by shook-swarm in June. The 1.5 kg swarms of honey bees were placed in regular 10-frame Dadant hives for nomadic beekeeping, with six frames of organic wax foundation (Il Pungiglione Soc. Coop.). The swarms were provided with newly introduced sister queens and sugar syrup (Apiinvert®, Südzucker). The hives were arranged in a single row. After seven days, the new colonies were treated with 50 mL of 3.5% (w/v) oxalic acid dihydrate sucrose solution, trickled in between the frames to control the parasitic mite *Varroa destructor* [30].

### Yeast materials and cultivation

Three yeasts were isolated at the Laimburg Research Centre in South Tyrol, Italy, from *D. suzukii*-infested grapes in South Tyrol, Italy, during a preceding study by Bellutti et al. (2018) [20]: *Saccharomycopsis vini* strain LB-NB-1.33 (accession number: KP298011, abbreviation: Sv 1.33) and *Hanseniaspora uvarum* strain LB-NB-1.21 (accession number: KP298009, abbreviation: Hu 1.21), strain LB-NB-2.2 (accession number: MK567898, abbreviation: Hu 2.2), and strain LB-NB-3.4 (accession number: MK567905, abbreviation: Hu 3.4). *Saccharomyces cerevisiae* (strain S288c, abbreviation: Sc S288c) is a conventional laboratory strain. For long-term storage, purified isolates were cultivated in chloramphenicol yeast glucose broth (5 g/L yeast extract, 20 g/L glucose, and 0.1 g/L chloramphenicol) and stored in 20% glycerol at -80°C. Yeasts were cultured in 220 mL of potato dextrose broth (PDB) (24 g/L Difco™ Potato Dextrose Broth) at 25°C, 120 rpm for 30 h in a 300-mL Erlenmeyer flask closed with cotton and aluminum foil [28].

### Plant materials and cultivation

Leaves from strawberry plants (*Fragaria x ananassa*, cultivar Elsanta) and grape vines (*Vitis vinifera,* cultivar Vernatsch -also known as Trollinger or Schiava, clone: Edelvernatsch Lb 43, Rootstock: SO4) were used in behavioural experiments. Plants were grown between May and July in the greenhouse under controlled conditions (22 ± 2°C, 75 ± 5% relative humidity, without artificial light) and treated once a week for 20 min with vaporised sulphur against powdery mildew using a sulphur burner (Nivola B.V. 220V, Holland). No sulphur treatments were performed during the assays. Leaves were selected to be similar in size and colouration for both treatment and control samples. Purchased blueberries (*Vaccinium corymbosum)* from organic production and various seasonal cultivars were used as substrates for oviposition experiments.

### Four-choice arena assay

To evaluate the preference of *D. suzukii* when multiple yeasts are available, the yeasts *S. vini,* strain 1.33 and *H. uvarum*, strains 1.21, 2.2 and 3.4 were presented in a competitive arrangement in a four-choice set-up (S1A Fig) [31]. For this purpose, five round glass dishes (diameter: 115 mm, height: 64 mm) were closed with a thin mesh. Each dish contained four traps, made of 4-mL glass vials that were closed with cut pipette tips that allowed the flies to get into the vials but prevented them from leaving. Each vial contained 1 mL of one of the four liquid yeast cultures. Groups of 20 females were placed into each dish and the number of females trapped was scored after 24 h.

The *H. uvarum* strain 2.2 was selected among the three *H. uvarum* strains for the rest of the study as there was no preference among them. Additionally, *H. uvarum* strain 2.2 was previously shown to be highly attractive to and to elicit electrophysiological response in *D. suzukii* [29].

### Flight attraction in wind tunnel

To evaluate odour-mediated attraction of *D. suzukii* in response to yeast headspace volatiles, wind tunnel experiments were conducted using a slightly modified protocol from Reherman et al. (2022) (S1B Fig) [26]. For each yeast tested, 25 mL of a yeast culture grown in PDB for 24 h was poured into a wash bottle. The stimulus was delivered in charcoal-filtered air (0.3 L/min) that was blown through the wash bottle containing the odour source. The scented airstream was vertically injected at the upwind end into the wind tunnel onto an 18 cm high, 38 mm diameter horizontal platform of aluminium, from which it diffused downwind as an odour plume. Four-to-six days old adult females were starved for 24 h prior to testing. They were transferred individually to a 30-mL glass tube and released at the down-wind end of the tunnel and exposed for 3 min to the main air stream (0.3 m/s) carrying a plume of yeast odours. Mated individual females were tested to see if they responded to the volatiles released by *H. uvarum* (n = 32), *S. vini* (n = 32), *S. cerevisiae* (n = 42), or PDB as a control (n = 48). The number of flies performing an upwind flight towards the scented air was counted.

### Field trapping

Trapping trials were performed in July 2020 at a forest edge in Laimburg (46°22’43.2“N 11°17’06.1”E, South Tyrol, Italy). The forest was a deciduous forest containing cherry and elderberry as host plants. The neighbouring crops included cherries, which were harvested shortly before the experiment. The traps consisted of 4-mL transparent glass vials filled with 2 mL PDB or culture of *S. vini, H. uvarum* or *S. cerevisiae*, respectively. To reduce the surface tension, 2 µL of Tween 20 per mL were added. The traps were fixed with a wire at a height of 1.5 m on branches, placed 3 m from each other and randomised. After 24 h, the traps were replaced to avoid non-experimental and undesired growth of microorganisms, then newly randomised. The number of trapped *D. suzukii* was counted. Each treatment was replicated 24 times.

### Mortality and oviposition assays

Spinosad (Laser, Corteva AgriscienceTM, Milan, Italy) was selected as a suitable insecticide [26]. Four treatments were created as follow: (1) water, (2) water with spinosad, (3) *S. vini* + spinosad and, (4) *H. uvarum* + spinosad. For the samples containing Spinosad, 5.43 mg spinosad per L liquid was added. In each trial, single grape or strawberry plants were considered as replicates. Ten leaves per plant were treated with 10 drops of 10 mL using a multichannel pipette in the greenhouse. Two experiments were performed: one on the first day, and one 7 days following the treatment. Five leaves belonging to one plant were removed and placed in an insect cage with their stems inserted into an Erlenmeyer flask filled with water. The opening around the stems was closed with cotton. A 5% (w/v) sugar solution supplied on cotton in a small Petri dish (diameter 6 cm) served as a water and energy source for the flies. Each cage also contained a Petri dish with water agar (diameter 9 cm, 15 g/L agar) on which four well washed blueberries were placed. Groups of 20 males and 20 females *D. suzukii* were then inserted. The blueberries and the agar substrates were removed and replaced by a new set 24 h after the start of the experiment. After 48 h, egg-laying was quantified from the number of eggs laid on the agar and berries and adult mortality was assessed. Five replicates were performed for each treatment.

### Attraction assay with *Apis mellifera*

The attractiveness of yeast cultures was assessed on honey bee workers. Experiments were performed near the hives, in September, a season with no major flower blossoming [32]. At least 30 min before each experiment, bees were trained on a plastic board placed 2 meters in front of the hives by providing sugar syrup and honey. A 5% (w/v) sugar water syrup was made fresh and added to the cultures of *S. vini*, *H. uvarum* and PDB. Three experimental designs were created: (i) the syrup and yeasts were presented on the same plate, (ii) the syrup and yeasts were presented on separate plates, (iii) the syrup and yeasts were presented in separate plates and dried at 35°C prior to the presentation. The dried conditions represented the yeast cultures after application on leaves. The PDB and sugar water syrup were tested with a similar sugar content to the yeast cultures [28]. The plates were randomly rotated every 5 min. The number of bees reaching each plate over a period of 5 min was assessed. Experiments lasted 80 min and were video recorded using an HD Camera (COAU Action, 4k 20mp) and VLC Media Player 3.0.11 (VideoLAN software).

### Yeast headspace characterisation by SPME-GC-TOF-MS

Solid phase microextraction followed by gas chromatograph-time of flight-mass spectrometry (SPME-GC-TOF-MS, QP2010 SE Shimadzu) was used to characterise volatile organic compounds (VOCs) from yeast headspaces, following the methodology of Alves *et al.* [33]. For each sample (yeast cultures and PDB, six replicates per sample), 5 mL were transferred to a 20-mL vial and 1 g of NaCl (99.0%) was added. The vial was capped with a polytetrafluoroethylene (PTFE) septum and an aluminium screw cap, and the metabolic quenching was achieved by freezing the samples at -80°C. Strict control of the quenching procedure, which arrests the cellular metabolism and enzymatic reactions of yeast, was applied to reduce data variability.

Sample analysis was randomised, and pooled and blank samples were injected after every ten samples for quality control and normalisation. The thawed samples were held in the autosampler at 15°C, incubated at 40°C for 15 min, and extracted with a divinylbenzene-carboxen-polydimethylsiloxane (CAR/DVB/PDMS) fiber 50/30 μm for 45 min. Desorption was performed at 250°C for 2 min. The GC instrument was operated in spitless mode using helium as the carrier gas with 1 mL/min, and the separation was achieved on a ZB-WAX column (30 m x 0.25 mm ID, 0.25 µm thickness, Phenomenex). A gradient temperature programme was used: 35°C for 5 min, then increased linearly from 35°C to 250°C at a rate of 3°C/min, then held at 250°C for 5 min. The transfer line and ion source temperatures were set at 250°C, with the ion source voltage at 70 eV. Mass spectrometric data were acquired in full scan mode over an *m/z* range of 40–510.

MS-DIAL was used for deconvolution, peak picking and alignment [34]. A total of 564 features were extracted based on peak height. After blank subtraction and combination of fragments of the same peak, 217 compounds were identified. To characterise each compound, the mass spectra were compared with mass spectral libraries, including an in-house library of standards, the NIST 2017 database and retention indices. The experimental linear retention index (LRI) of each compound was calculated using a series of *n*-alkanes (C8-C20) under the same experimental conditions. In total, 156 compounds were annotated, each presenting similarity matches > 800 with the libraries and LRI-matches.

### Yeast headspace volatile detection in EAG

Electroantennography (EAG) experiments were conducted to identify which chemicals are detected by adult *D. suzukii*. In each experiment, a ﬂy was immobilised in a truncated plastic pipette tip, with half of its head protruding from the narrow end. Antennal activity was measured by placing a recording electrode over the tip of the antenna and an indiﬀerent electrode near the base of the antenna (through the eye). Ag–AgCl glass electrodes were ﬁlled with Beadle–Ephrussi Ringer solution [35].

Signals were passed through a high-input impedance ampliﬁer (2-channel USB acquisition controller, IDAC-2; Syntech) and recorded using GC-EAD 2014 software (v1.2-5, Syntech). An air pulse (stimulus) lasting 3 s was delivered through a cartridge into a carbon-ﬁltered and humidiﬁed air stream directed at the ﬂy preparation.

The cartridges consisted of a glass Pasteur pipette (ThermoFisher Scientiﬁc) with a ﬁlter paper (15 mm diameter, Whatman grade 1) placed within the larger end and closed with a 1-mL pipette tip. A 30 µL aliquot of a 1000-fold diluted chemical in paraﬃn oil (10^-3^ v/v) was deposited onto the filter paper just before the experiment. A cartridge containing only paraﬃn oil served as a solvent control. A positive control, using 2-heptanone, was used to correct for eventual antennal fatigue and to standardise the responses across replicates. This compound is known to elicit antennal responses in *D. suzukii* [36]. Each cartridge was used for a maximum of three stimulations to prevent significant changes in chemical concentration. All chemicals used in the experiments were purchased with the highest purity available (S2 Appendix).

### Yeast headspace volatile detection in GC-EAD

Coupled gas chromatography-electroantennography detection (GC-EAD) was used to elute each pure chemical onto the antenna and identify any antennally active contaminants present in the standard solutions tested. In this setup, the electroantennography detector (described above) was coupled with a GC (7820A, Agilent Technologies) equipped with a flame ionisation detector (FID). The fly was prepared as described above.

Standards were diluted in dichloromethane and organised into four mixtures, each containing several compounds at a 10^-3^ v/v dilution. A 3 µL aliquot of each mixture was injected into the GC column (HP-5MS Agilent 19,091 J-413 column, 0.25 μm coating 30 m length and 0.32 mm diameter) through a cool-on-column (COC) injector. Helium, at a ﬂow rate of 2.5 mL/min, was used as the carrier gas. The oven method was programmed as follows: inject at 50°C and hold for 1.8 min, then 7.3°C/min to 250°C and hold for 3 min. The injector temperature was set at 250°C and the detector temperature was set at 350°C. The column effluent was mixed with a nitrogen make-up and split at a 1:1 ratio. One portion ﬂowed to the FID, while the other portion was directed through a transfer line at 170°C into a charcoal- ﬁltered and humidiﬁed airstream over the mounted fly. Signals were ampliﬁed via an EAG ampliﬁer (as described above). GC-FID and EAD signals were simultaneously recorded using GC-EAD 2014 software (v 1.2-5, Syntech).

### Data analysis

Statistical analyses were performed using R 4.4.1. (R Core Team, 2024). A significance level of *P* = 0.05 was used for all comparisons. A generalised linear mixed model (GLMM, package `lme4’) fitted with a Poisson error distribution was applied to evaluate the distribution of flies accross the four traps ine the arena, and the number of trapped *D. suzukii* flies per vial in field trapping experiments.

The upwind flight attraction of yeast odours in the wind tunnel was analysed using a GLMM fitted with a binomial error distribution. The mortality rate of *D. suzukii* and the number of eggs laid (oviposition) per cage were evaluated using a generalised linear model fitted with a gamma distribution. To handle zero values and to allow the use of a gamma distribution, datasets were transformed using x + 1. Model selection was based on Akaike information criterion (AIC) values, and residuals were examined to verify the distribution of the errors. Treatment and fly sex (where applicable) were included as fixed effects. Post hoc comparisons were performed using Tukey’s contrast pairwise test (package ‘multcomp ’[37]).

The number of honey bees on feeding plates was analysed using linear mixed models (package `lme4’ [38]). Bee counts were considered as the response variable, while the tested solution, the position on the board, and time were considered as predictors. The rounds (i.e., the intervals during which the tested solutions were rotated on the board) were accounted as random effects. Model fitting was evaluated through residuals analysis (package `DHARMa’ [39]). Post-hoc comparisons between solutions were conducted using pairwise comparisons (`multcompview’ package [40]). Type II ANOVA tables (Anova function, `car’ package [41]) were generated to summarise the significance of fixed effects in the mixed-effects model.

The headspace composition was analysed as follows: peak height results were normalised based on the systematic error removal using the random forest (SERRF) method to remove systematic errors [42]. To identify significantly impacted lipid clusters among yeasts, a chemical similarity enrichment analysis (ChemRICH) was performed on the 156 annotated compounds identified, using the ChemRICH platform. This platform clusters significantly impacted metabolites based on chemical similarity and ontology mapping to highlight biologically relevant patterns. Next, a Kolmogorov–Smirnov test was used for subsequent statistical analysis [43].

The EAG and EAD signals were integrated using GC-EAD 2014 v1.2-5 (Syntech). Responses were measured as the maximum voltage deflection following the start of the stimulus. In EAG experiments, the antennal response to the solvent control was subtracted from the response to each standard, and the resulting values were normalised to the response elicited by the positive control (2-heptanone), which was set to 100%. The distribution of response amplitudes was assessed using a Shapiro-Wilk normality test. Subsequently, responses to the standards were compared to those elicited by the solvent control using two-sided paired t-tests (`stats’ package [44]). Standards that elicited a response signiﬁcantly greater than paraﬃn oil were considered antennally active. In GC-EAD experiments, a standard was considered antennally active if at least three flies displayed a non-zero voltage deflection. For both EAG and GC-EAD experiments, only flies that responded to the positive control were included in the analysis (n = 6–10).

## Results

### *Saccharomycopsis vini* and *Hanseniaspora uvarum* are attractive in long and short-range behavioural experiments

In the first behavioural assay, *S. vini* and three strains of *H. uvarum* were simultaneously presented to *D. suzukii* for 24 h in a 4-choice arena to evaluate their preference (Fig 3A, S1 Fig). Baits containing *S. vini* were significantly more attractive than those containing *H. uvarum* 1.21 (GLMM Poisson distribution, MCM: *X*^2^_3** **_= 25.72, *z* = 3.72, *P* = 0.001), *H. uvarum* 2.2 (*z* = 3.85, *P* < 0.001) and *H. uvarum* 3.4 (*z* = 3.20, *P* = 0.008). The three *H. uvarum* strains were not different from each other (*P* > 0.05). Notably, 2% of the flies tested did not choose any yeast bait.

**Fig 1:**
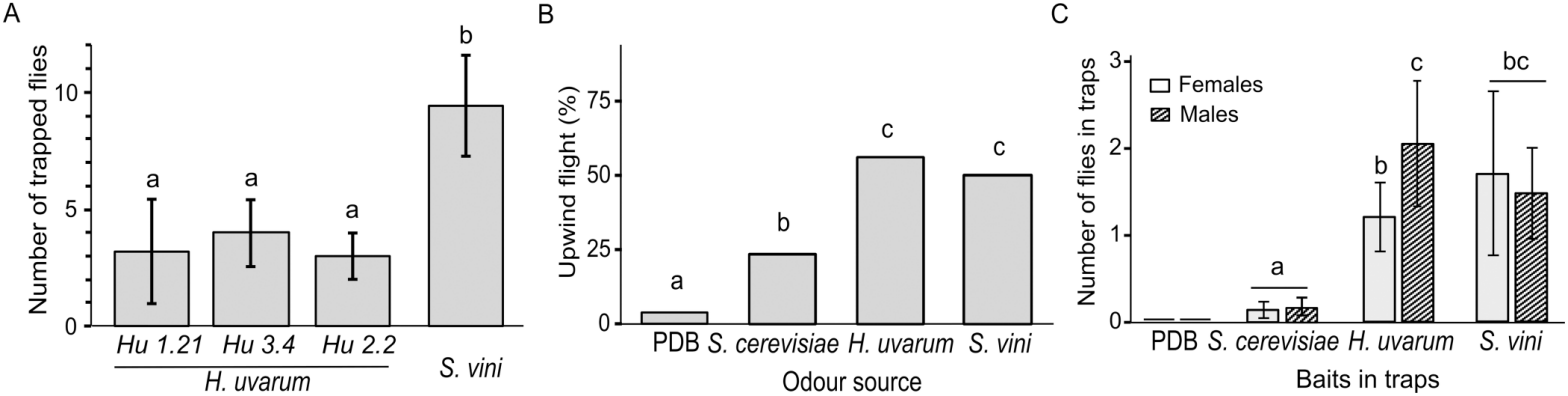
Short- range and long range attraction assays. (A) Mean (± SD) number of female *Drosophila suzukii* trapped in four simultaneously presented baits within 24 h. Baits consisted of yeast culture of *Hanseniaspora uvarum* and *Saccharomycopsis vini* in potato dextrose broth (PDB). Three strains of *H. uvarum*: Hu 1.21, Hu 2.2 and Hu 3.4 were tested. Bars with different letters are significantly different (GLMM Poisson distribution and multiple comparison of means, *P* < 0.05). B) Percentage of females flying upwind towards headspace volatiles of *H. uvarum* (Hu 2.2), *S. vini*, *Saccharomyces cerevisiae* and PDB in a wind tunnel. Bars with different letters are significantly different (GLM binomial distribution and multiple comparison of means, P < 0.05). C) Mean (± SD) number of females (plain bars) and males (striped bars) caught in traps placed on trees at a forest edge. Traps were baited with 2 ml culture of *H. uvarum* (Hu 2.2), *S. vini*, *S. cerevisiae* or PDB. Bars with different letters are significantly different (GLMM, Poisson distribution, *P* < 0.05).

**Fig 2:**
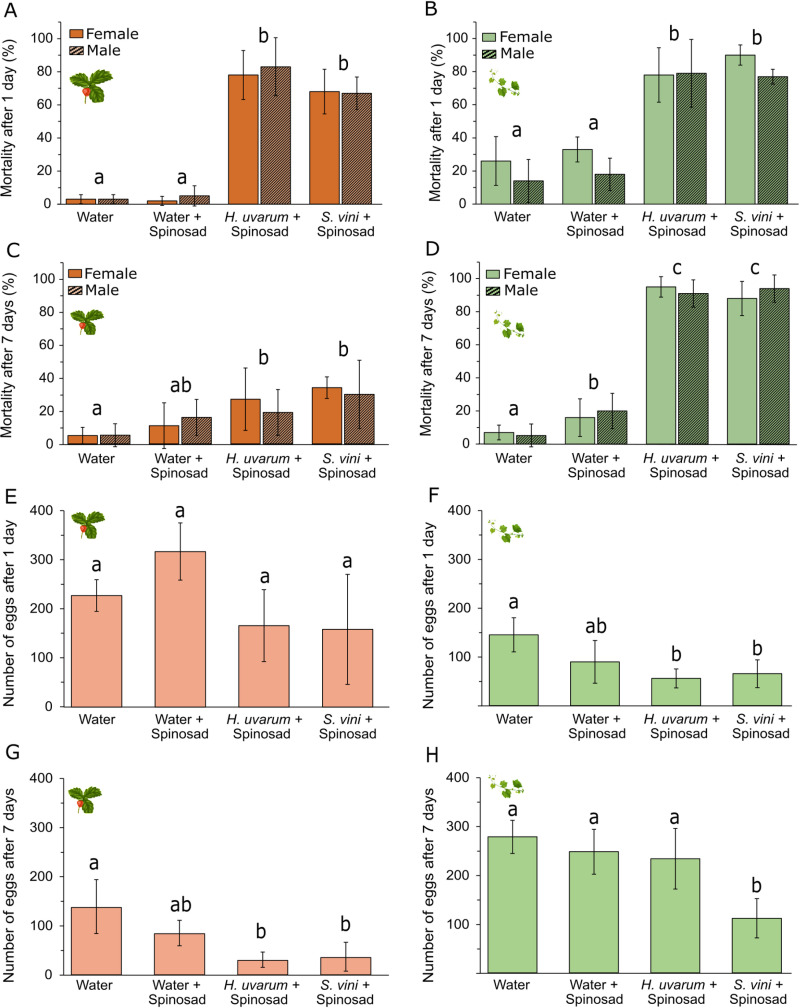
Mortality and oviposition assays with attract-and-kill formulation. (A–D) Mean (± SD) mortality rate (%) of female (‘plain’ bars) and male (‘stripe’ bars) *Drosophila suzukii* exposed to treated strawberry (red bars, panels A and C) or grapevine (green bars, panel B and D) leaves one day after treatment (panels A and B) and seven days after treatment (panels C and D). (E–H) Mean number (±SD) of eggs laid during a 48 h exposure to treated strawberry (red bars, panels E and G) or grapevine leaves (green bars, panels F and H) one day after treatment (panels E and F) and seven days after treatment (panels G and H). Leaves were treated with formulations of spinosad with water, *Hanseniaspora uvarum*, or *Saccharomycopsis vini*. A water treatment served as a control. Bars with different letters are significantly different (GLM and Tukey´s multiple comparison, *P* < 0.05).

**Fig 3:**
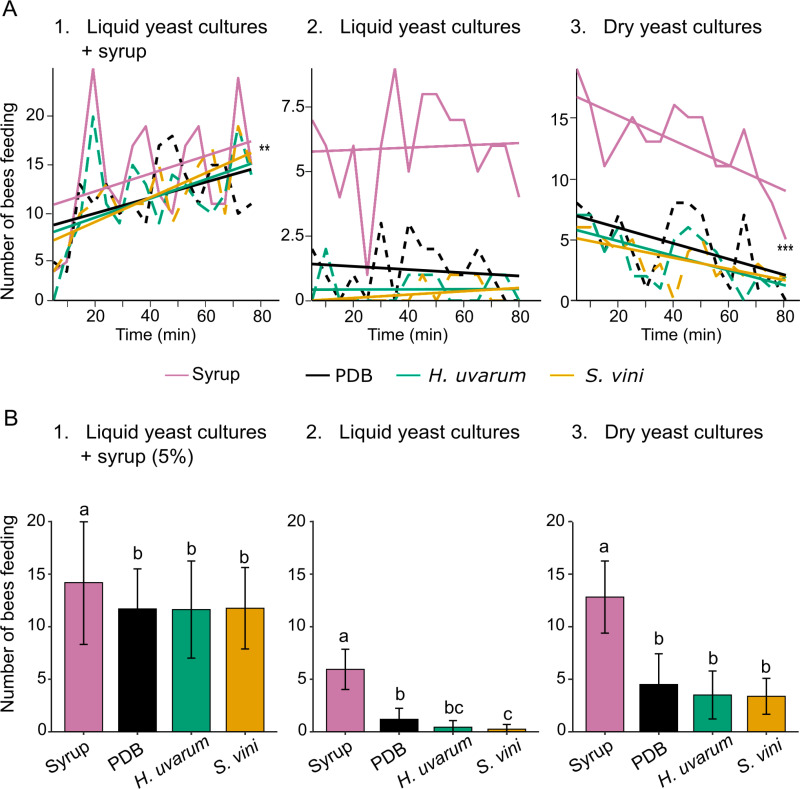
Attraction assay with *Apis mellifera.* (A) Total number of *Apis mellifera* foragers feeding on four plates simultaneously presented, containing 5% sugar syrup, potato dextrose broth (PDB) growth medium, and yeast cultures of *Hanseniaspora uvarum* and *Saccharomycopsis vini* over a period of 80 minutes in three separate experiments where plates contained: 1) liquid yeast cultures + sugar syrup 5%; 2) liquid yeast cultures; 3) dry yeast cultures. The straight lines report the linear trend calculated as y = ax + b. Asterisks indicate statistical significance (** *P* < 0.01, *** *P* < 0.001) of the time on the number of feeding bees. B) Mean (± SD) number of bees feeding from the four plates over 5 min. Different letters report a statistical difference between treatments (LMM, and pairwise multiple comparison, *P* < 0.05). Foragers from five colonies of similar strength were tested. Sixteen observations of 5 min each were performed 30 min after training with sugar syrup.

The second behavioural assay, evaluated the flight attraction of *D. suzukii* females to yeast odours in a wind tunnel. During a 3-min test period, flies were exposed to each yeast, placed upwind (Fig 1B, S1 Fig). Flies displayed flight behaviour and were similarly attracted to *S. vini* and *H. uvarum* (GLM binomial distribution: *X*^2^_3_ = 36.85, *z* = 0.50, *P* = 0.62). Both yeasts were significantly more attractive than *S. cerevisiae* (*z* = ‐2.30, *P* = 0.02). The control (PDB) elicited significant less upwind flight than any of the three yeasts (*P* < 0.05).

In a third experiment, traps containing small volumes (2 ml) of either *S. vini, H. uvarum* or *S. cerevisiae* cultures, were placed on a forest edge to evaluate their attractiveness to wild *D. suzukii* populations (Fig 3C). Traps baited with *H. uvarum* and *S. vini* captured significantly more flies than those baited with *S. cerevisiae* (GLM, Poisson distribution, *F*_2,141_ = 193.24, *S. vini*: *z* = 4.959, *P* < 0.001; *H. uvarum*: *z* = 5.106, *P* < 0.001). There was no difference in the number of flies captured in the baits with *S. vini* and *H. uvarum* (*z* = 0.293, *P* = 0.952). No flies were found in traps containing PDB. Notably, traps baited with *H. uvarum* captured more males than females (*F*_1,140_ = 4.800, *P* = 0.028) whereas no sex bias was observed in traps baited with *S. vini.*

### Attract-and-kill formulations with *S. vini* and *H. uvarum* effectively reduced oviposition and increased mortality

On strawberry leaves, one day after treatment the mortality was significantly impacted (*F*_3,36_ = 53.653, *P* < 0.001, Fig 2A). Mortality in the water control was significantly lower compared to spinosad formulations with *H. uvarum* (*z* = 4.763, *P* < 0.001) and *S. vini* (*z* = 4.714, *P* < 0.001). Mortality was also significantly lower in spinosad with water compared to formulations containing *S. vini* (*z* = 4.675, *P* < 0.001) and *H. uvarum* (*z* = 4.731, *P* < 0.001). Oviposition was not significantly affected after exposure to the three formulations or water (*F*_3,16_ = 2.862, *P* = 0.069, Fig 2E).

Seven days after the application, a significant effect on the mortality was observed (*F*_3,36_ = 8.507, *P* < 0.001, Fig 2C). Mortality in the water control remained significantly lower compared to spinosad formulated with *H. uvarum* (*z* = 3.077, *P* = 0.009) and *S. vini* (*z* = 3.412, *P* = 0.003). Oviposition was also significantly affected (*F*_3,16_ = 8.390, *P* = 0.001, Fig 2F) and was significantly higher in the water control compared to spinosad formulations with *S. vini* (*z* = 2.880, *P* = 0.017) and *H. uvarum* (*z* = 3.079, *P* = 0.009).

On grapevine leaves, one day after treatment and after 48 h exposure mortality was significantly affected (*F*_3,36_ = 27.209, *P* < 0.001, Fig 2B). Mortality in the control was significantly lower compared to spinosad with *H. uvarum* (*z* = 5.293, *P* < 0.001) and *S. vini* (*z* = 5,426, *P* < 0.001). Furthermore, the mortality with spinosad in water was significantly lower compared to formulations containing *S. vini* (*z* = 4.872, *P* < 0.001) and *H. uvarum* (*z* = 4.705, *P* < 0.001). Oviposition was also significantly affected (*F*_3,16_ = 6.105, *P* = 0.006, Fig 2G). Oviposition in the control was significantly higher compared to spinosad with *S. vini* (*z* = 2.873, *P* = 0.019) and *H. uvarum* (*z* = 3.296, *P* = 0.005).

Seven days after the application, the mortality was also significantly affected (*F*_3,36_ = 54.181, *P* < 0.001, Fig 2D). Mortality in the control was significantly lower compared to the one following exposure to spinosad formulated with *H. uvarum* (*z* = 6.008, *P* < 0.001), *S. vini* (*z* = 5.996, *P* < 0.001) and water (*z* = 3.824, *P* < 0.001). Mortality following exposure to spinosad with water was also significantly lower compared to spinosad with *H. uvarum* (*z* = 5.097, *P* < 0.001) and *S. vini* (*z* = 5.064, *P* < 0.001), which were not significantly different from each other (*z* = 0.099, *P* = 1). Oviposition was also significantly affected (*F*_3,16_ = 12.018, *P* < 0.001, Fig 2H). Oviposition after exposure to spinosad with *S. vini* was significantly lower compared to spinosad with water (*z* = 4.489, *P* < 0.001), with water control (*z* = 4.979, *P* < 0.001) and *H. uvarum* (*z* = 4.216, *P* < 0.001).

### Yeast cultures did not stimulate foraging of *Apis mellifera*

The attractiveness of yeast VOCs and sugar content was assessed using trained honey bees to determine whether the application of yeast cultures would interfere with their foraging behaviour (Fig 3). Across all three experimental conditions and throughout the 80-minute trial, a 5% sugar syrup consistently proved more attractive than yeast cultures or PDB: (1) when syrup was presented alongside yeast cultures (LMM, *χ*²_₃,₆₃_ = 17.98, *P* < 0.01), (2) when syrup and liquid yeast cultures were presented separately (LMM, *χ*²_₃,₆₃_ = 149.17, *P* < 0.001), and (3) when syrup and dried yeast cultures were presented separately (LMM, *χ*²_₃,₆₃_ = 140.87, *P* < 0.001). Time had a significant effect in two conditions: (1) when syrup was presented with the cultures (LMM, *χ*²_1,63 _= 170.81, *P* < 0.01), showing an overall increase of visitation (bee counts) and (3) when syrup and dried yeast cultures were presented separately (LMM, *χ*²_1,63 _= 32.82, *P* < 0.001), where visitation decreased over the course of the trial.

### Yeasts have distinct and overlapping headspace compositions in SPME

We characterised 156 VOCs by SPME-GC-TOF-MS and performed a ChemRICH analysis to statistically compare the presence and abundance of each compound between *S. vini*, *H. uvarum* and *S. cerevisiae* (S2 Table). From this analysis we extracted classes of chemicals that significantly differed between the yeasts (S3 Table). The headspace compositions of *S. vini* and *H. uvarum* were significantly different in alcohols and polyols, benzene and substituted derivatives, branched unsaturated hydrocarbons, carboxylic acid esters, fatty acid esters, fatty alcohols, ketones and monoterpenoids (FDR-adjusted Kolmogorov–Smirnov-test, *P* < 0.01). Comparing *H. uvarum* and *S. cerevisiae*, significant differences were found in alcohols and polyols, benzene and substituted derivatives, carboxylic acid esters, fatty acid esters, ketones, medium-chain fatty acids, monoterpenoids and sesquiterpenoids (FDR-adjusted Kolmogorov–Smirnov-test, *P* < 0.01). *Saccharomycopsis vini* and *S. cerevisiae* headspace compositions also differed significantly in alcohols and polyols, benzene and substituted derivatives, branched unsaturated hydrocarbons, carboxylic acid esters, fatty acid esters, fatty alcohols, ketones, medium-chain fatty acids, monoterpenoids and sesquiterpenoids (FDR-adjusted Kolmogorov–Smirnov test, *P* < 0.01).

Within these classes, 40 compounds differed significantly between the yeasts (Table 1). Specifically, *S. vini* produced significantly higher amounts of 14 monoterpenoids and 4-methyl-1-pentanol. *H. uvarum* produced the largest amount of 2-phenethyl acetate, isobutyl acetate, 2-methylbutyl acetate, 2-nonanol, acetoin and (6*E*)-nerolidol. Both *H. uvarum* and *S. vini* produced significantly higher amounts of ethyl acetate, ethyl propanoate and 2-acetylthiazole compared to *S. cerevisiae.* Conversely, *S. cerevisiae* produced the largest amounts of 3-methyl-1-butanol, 2-phenylethanol, 2-methylthiolan-3-one, 1-heptanol, ethyl octanoate, hexanoic acid, octanoic acid, nonanoic acid and decanoic acid.

**Table 1:** Mean amounts of headspace compounds in *Hanseniaspora uvarum*, *Saccharomycopsis vini* and *Saccharomyces cerevisiae,* antennal response amplitudes in EAG, and percentage of *Drosophila suzukii responding* in GC-EAD.

	Compound^1^	Mean ± SD peak height								Mean ± SD amplitude^2^		Responses (%) in GC-EAD^4^
	*H. uvarum*		*S. vini*			*S. cerevisiae*					
**Alcohols and polyols**												
	3-Methyl-1-butanol	1140713 ± 307794	^a^		1434013 ± 225180	^b^		2309561 ± 836363	^b^	-24.12 ± 22.68	*	100
	(R,R)-2,3-Butanediol	171 ± 56	^a^		1391 ± 948	^b^		2132 ± 1060	^b^	-29.67 ± 30.66		0
	4-Methyl-1-pentanol	359 ± 36	^a^		2345 ± 681	^b^		395 ± 54	^a^	-29.67 ± 22.85	*	100
	3-Methyl-1-pentanol	590 ± 68	^a^		1077 ± 184	^b^		1703 ± 546	^c^	-11.94 ± 6.28		100
**Benzene and substituted derivatives**												
	Toluene	565 ± 419	^a^		1534 ± 617	^b^		1420 ± 383	^b^	-15.13 ± 15.02		100
	2-Phenylethanol	582979 ± 130899	^a^		753122 ± 170800	^a^		1170504 ± 142925	^c^	-6.62 ± 7.18		0
	2-Phenylethyl acetate	89529 ± 60121	^a^		5177 ± 4161	^b^		30176 ± 5580	^a^	-39.95 ± 30.34		0
**Branched unsaturated hydrocarbons**												
	ʏ-Terpinene	85 ± 21	^a^		2047 ± 1153	^b^		70 ± 18	^a^	0.12 ± 0.14	*	0
**Carboxylic acid esters**												
	Ethyl acetate	1747424 ± 265873	^a^		1217426 ± 598476	^a^		33251 ± 2046	^c^	-60.46 ± 26.21	*	100
	Ethyl propanoate	3330 ± 1689	^a^		12562 ± 3900	^a^		1020 ± 99	^c^	-36.7 ± 42.23	*	100
	Isobutyl acetate	15486 ± 3650	^a^		947 ± 234	^b^		2105 ± 858	^c^	-30.1 ± 19.57	*	100
	2-Methylbutyl acetate	295741 ± 101587	^a^		11027 ± 2266	^b^		60878 ± 7239	^c^	-27.26 ± 15.6	*	100
**Fatty acid esters**												
	Ethyl butanoate	360 ± 74	^a^		1528 ± 597	^b^		1358 ± 298	^b^	-50.14 ± 16.66	*	100
	Ethyl octanoate	7205 ± 2609	^a^		2678 ± 969	^b^		245010 ± 64693	^c^	0.1 ± 0.15	*	0
**Fatty alcohol**												
	1-Heptanol	11808 ± 4873	^a^		2545 ± 522	^b^		126349 ± 32068	^c^	-13.63 ± 14.99		0
	2-Nonanol	3160 ± 263	^a^		634 ± 178	^b^		406 ± 109	^c^	-8.61 ± 11.68		0
**Ketones**												
	Acetone	2936 ± 541	^a^		4671 ± 1217	^b^		4760 ± 517	^b^	-20.02 ± 22.29		0
	Acetoin	42568 ± 12698	^a^		2059 ± 2082	^b^		10715 ± 3780	^c^	-51.09 ± 48.21	*	100
	2-Methylthiolan-3-one	896 ± 78	^a^		221 ± 84	^b^		1557 ± 251	^c^	-13.83 ± 13.29	*	100
	2-Acetylthiazole	1558 ± 106	^a^		1591 ± 125	^a^		691 ± 174	^c^	-5.08 ± 9.28		0
	2-Undecanone	277 ± 37	^a^		2470 ± 534	^b^		591 ± 136	^c^	1.14 ± 9.82		0
**Medium-chain fatty acids**												
	Hexanoic acid	1919 ± 343	^a^		2490 ± 741	^a^		46736 ± 14569	^c^	-13.94 ± 16.65		0
	Octanoic acid	6973 ± 1645	^a^		1383 ± 1383	^b^		100883 ± 32045	^c^	-9.19 ± 27.81		0
	Nonanoic acid	1264 ± 466	^a^		579 ± 271	^b^		2795 ± 1016	^c^	3.95 ± 6.12	*	0
	Decanoic acid	876 ± 287	^a^		574 ± 645	^a^		42893 ± 12266	^c^	-10.14 ± 16.53		0
**Monoterpenoids**												
	β-Myrcene	201 ± 333	^a^		176450 ± 83832	^b^		133 ± 105	^a^	-0.48 ± 0.87		30
	Limonene	97 ± 67	^a^		23163 ± 11489	^b^		129 ± 88	^a^	-7.76 ± 14.31		0
	p-Cymene	163 ± 75	^a^		8290 ± 4034	^b^		89 ± 32	^a^	-17.33 ± 30.6		0
	(*Z*)-β-Ocimene^+^	115 ± 94	^a^		76852 ± 37254	^b^		81 ± 26	^a^	-0.51 ± 0.36		0
	(*E*)-β-Ocimene^+^	150 ± 135	^a^		114930 ± 58883	^b^		85 ± 49	^a^	-0.51 ± 0.36		0
	Linalool	1189 ± 427	^a^		195699 ± 86629	^b^		590 ± 323	^c^	-1.63 ± 1.32		40
	(4*Z*,6*Z*)-Allocimene^+^	94 ± 17	^a^		20701 ± 8883	^b^		86 ± 14	^a^	-0.88 ± 0.96	*	0
	α-Terpineol	237 ± 55	^a^		12503 ± 5776	^b^		407 ± 92	^c^	-5.2 ± 12.15		0
	(*Z*)-Geraniol	922 ± 429	^a^		126173 ± 63976	^b^		2476 ± 567	^c^	-3.18 ± 5.49	*	0
	Citronellol	1272 ± 534	^a^		178389 ± 80138	^b^		1110 ± 299	^a^	-11.74 ± 15.15		0
	Nerol	7863 ± 1791	^a^		1669510 ± 511392	^b^		3669 ± 870	^c^	-10.23 ± 12.48		0
	Geranial	523 ± 136	^a^		46115 ± 18434	^b^		341 ± 115	^c^	-9.51 ± 13.89		0
**Sesquiterpenoids**												
	(*E*,*Z*)-ɑ-Farnesene^+^	817 ± 107	^a^		691 ± 701	^a^		435 ± 212	^a^	-2.73 ± 4.29		0
	(6*E*)-Nerolidol	1809 ± 794	^a^		836 ± 168	^b^		1512 ± 883	^ab^	-7.15 ± 13.55		0
	(2*Z*,6*E*)-Farnesol^+^	80 ± 21	^a^		98 ± 33	^a^		332 ± 108	^b^	-5.26 ± 3.85		0

^1^Compounds identified as significantly different between the three yeasts species by solid phase microextraction followed by gas chromatograph-time of flight-mass spectrometry (SPME-GC-TOF-MS) and chemical similarity enrichment analysis. Mean ± SD peak height with different letters are significantly different (n = 6). ^+^ The standard solution included several isomers. ^2^ Mean ± SD amplitude (mV) of antennal response in electroantennography (EAG) recordings. * P < 0.05 (n = 6–10). ^3^ Percentage of antennal responses (n = 6–10) in gas chromatography antennography detection (GC-EAD). Standards were of dilution 10^−3^ v/v.

### Yeast headspace volatiles detection in EAG and GC-EAD

In EAG experiments, we tested whether *D. suzukii* could detect the 40 compounds released in significantly different quantities by the three yeast species (Table 1, S4 Table). Significant antennal responses were observed for 14 compounds (paired t-test, *P <* 0.05): 3-methyl-1-butanol, 4-methyl-1-pentanol, γ-terpinene, ethyl acetate, ethyl propanoate, isobutyl acetate, 2-methylbutyl acetate, ethyl butanoate, ethyl octanoate, acetoin, 2-methylthiolan-3-one, nonanoic acid, allocimene, and (*Z*)-geraniol.

Then, we also assessed whether these 40 compounds were antennally active in GC-EAD (Table 1, S5 Fig). Antennal responses (deflection < 0 mV) were observed in 100% of the tested flies for 11 compounds: 3-methyl-1-butanol, 4-methyl-1-pentanol, 3-methyl-1-pentanol, toluene, ethyl acetate, ethyl propanoate, isobutyl acetate, 2-methylbutyl acetate, ethyl butanoate, acetoin and 2-methylthiolan-3-one. In addition, responses were recorded in 4 out of 10 flies for linalool and β-myrcene.

## Discussion

Attract-and-kill strategies are efficient in various crop systems against many pest insects [45,46]. In this study, we evaluated the potential of *S. vini* to be used in attract-and-kill strategies to manage *D. suzukii* as this was done with *H. uvarum.* We also assessed how the two yeasts would affect bee foraging. Lastly, we identified antennally active chemicals from their headspaces.

Adding *S. vini*, as an attractant to spinosad significantly enhanced the efficacy of spinosad, nearly doubling it one week after application. This approach increased mortality rates and reduced oviposition in two crops, grapevine and strawberry, and was most comparable to the formulation of spinosad with *H. uvarum*. It is important to note that interactions between the yeasts and plants may create a complex chemical environment for the flies, as noted by Bruce and Pickett [47]. Despite their distinct chemical headspace profiles and metabolomic properties, both *S. vini* and *H. uvarum* effectively attracted *D. suzukii* [23,29]. Each yeast species and strain has unique specificities that warrant further investigation across different crop systems. For instance, using different *H. uvarum* strains, which are equally attractive, across diverse crop systems could provide valuable insights. This approach would be of significant value for the management of *D. suzukii*, especially considering that summer and winter phenotypes of *D. suzukii* display different levels of attraction to yeast baits [48,49]. Consequently, relying on a single strategy alone may not be effective throughout the year as *D. suzukii* is active for several seasons moving from host to host [2,50].

It is crucial to evaluate the interactions between integrated and organic pest management tools and non-target species such as pollinators [51–53]. Our findings indicate that the yeast cultures used in our formulations, whether presented in wet or dry form, were not preferred by honey bees. Some feeding occurred which may be triggered by prior training of the bees to the feeding site. These results are in agreement with previous studies on the attractiveness of yeast-contaminated food sources to honey bees and bumblebees [54–57]. Therefore, these attract-and-kill formulations are unlikely to interfere with honey bee foraging behaviour, making them promising approaches for further development in larger field trials. In addition, these strategies involve applying the formulations on leaves at the beginning of fruit production, in order to target the early development of *D. suzukii* populations and there by reducing subsequent infestations [58] and minimising the contact between the formulation and non-target species.

Our results demonstrated that *D. suzukii* preferred *S. vini* over three strains of the highly attractive *H. uvarum* when presented simultaneously. This finding aligns with previous research, indicating that *S. vini* is more attractive than other fruit-associated yeasts, including *H. uvarum* [29]. Notably, no differences were observed between the three *H. uvarum* strains, with strain Hu 2.2 being as attractive as in other works [26–29] in long and short range attraction experiments. Our study revealed that females were able to discriminate between both yeasts yet were equally attracted to both yeasts in long-range assays, while displaying a greater attraction to *S. vini* in short-range attraction assays. These two behaviours are important for designing attract-and-kill strategies, where long-range attraction is necessary to lure insects to a target site, but then close-range attraction triggers landing and perhaps feeding and/or oviposition [47,59]. Furthermore, flies were exposed to both static and dynamic airflow environments with diverse odour compositions, which could influence their attraction to yeasts. While in the first setting, flies were expected to choose from the available choices [60], in the second setting, they had only one available option located upwind. This highlights how environmental conditions impact yeast acceptance levels. For example, while *S. cerevisiae* has previously been shown to attract *D. suzukii* [21], it is less attractive compared to *S. vini* and *H. uvarum*. Our findings underscore the importance of conducting diverse behavioural experiments to gain a more thorough understanding of behavioural responses, thereby optimising strategies to exploit yeast preferences in pest management approaches.

To identify specific yeast volatiles that could be responsible for the discriminatory behaviour of *D. suzukii* towards *S. vini*, *H. uvarum* and *S. cerevisiae,* we characterised the headspace volatiles detected by *D. suzukii*. *Saccharomycopsis vini* and *H. uvarum* were distinguished by six yeast-specific compounds that elicited antennal responses: *H. uvarum* was characterised by isobutyl acetate, 2-methylbutyl acetate, and acetoin, while *S. vini* was characterised by 4-methyl-1-pentanol, Linalool and beta-myrcene. One or a mixture of these compounds could enable the fly to discriminate *S. vini* from *H. uvarum*. Furthermore, some compounds that did not differ in amount between yeasts were also reported as antennally active and associated with attraction in host-seeking *D. suzukii* [61–65]. The presence of these shared attractive compounds could explain why both yeasts exhibit similar levels of long-range attraction.

*Saccharomycopsis vini* had a unique headspace profile composed of 14 terpenes, compared to *H. uvarum*. Allocimene and (*Z*)-geraniol induced significant antennal responses in all flies in EAG experiments but were not detected in GC-EAD. Linalool and beta-myrcene were found to be antennally active, but only in a subset of the flies (0–40% instead of 100%). This result was also noted in Castellan et al. [29].

Many terpenes found in the headspaces of *S. vini*, such as linalool, nerol, beta-myrcene and limonene have been consistently reported to be antennally active in earlier studies [66] and specifically linalool was found to be detected [61,66–69] and attractive to *D. suzukii* [64,67,70,71]. Moreover, linalool and beta-myrcene are present in the headspace of ripening fruits like raspberries and blueberries, which are hosts for *D. suzukii* [61,72,73]. It is thus unclear why no responses to these chemicals were measured in GC-EAD in this study. Curiously, nerol and limonene were both found to be repellent to *D. suzukii* [66]. This appears contradictory with our results showing that *S. vini* is highly attractive while releasing high quantities of these compounds. Further behavioural study would clarify their relevance for *D. suzukii*.

In addition to yeast-specific attractive compounds, both *H. uvarum* and *S. vini*, produced the detected ethyl acetate and ethyl propanoate in significantly higher amounts compared to *S. cerevisiae.* These were found to be attractive to *D. suzukii* [64]. On the contrary, *S. cerevisiae* produced the largest amount of the antennally active 3-methyl-1-butanol. This yeast compound is attractive to *D. suzukii* [64,69,71,74] and therefore could be involved in the attractiveness of *S. cerevisiae* in the wind tunnel. 2-Methylthiolan-3-one was also higher in the least attractive yeast, *S. cerevisiae*, which corresponds with the findings of an earlier study [75]. We now found that this compound is being detected by *D. suzukii*, which warrants further investigation into its role.

We identified a greater number of headspace compounds compared to our previous work [29]. This increase can be attributed to modifications in the SPME method including an 10°C increase in incubation, and an additional 15 min of extraction time. Furthermore, the data were analysed using a metabolomic approach, which allowed for a comprehensive evaluation of compound profiles, rather than relying solely on pairwise comparisons based on average abundance [29,76,77]. These methodological changes underscore the need for multiple approaches to fully characterise yeast headspaces. Similarly, using both EAG and GC-EAD, we provided a more comprehensive analysis of chemical detection by *D. suzukii* [78–80]. With the GC-EAD method we obtained an antennal response to pure single chemicals of interest, thus eliminating impurities or a mixed effect in the case of geometric isomer mixtures. Although the EAG method carries a higher risk from unwanted chemical contaminations, it offered the advantage of a rapid quantification of the antennal response. We found differences between the two methods, where compounds were strongly antennally active in only one of the setups. Notably, the responses to 3-methyl-1-pentanol and toluene were visible in GC-EAD but not in EAG. Conversely, the responses to γ-terpinene, ethyl octanoate, nonanoic acid, allocimene and (*Z*)-geraniol were significant in EAG but absent in GC-EAG. *Drosophila suzukii* has been shown to detect these compounds in earlier works [4,69,70,81]. 3-Methyl pentanol was noted as an attractive foraging clue for *D. suzukii* [61,64], it is thus still unclear why it failed to induce a response in EAG. Furthermore, It was released by the three yeasts but in the highest amount in *S. cerevisiae*, the least attractive. The detection of toluene by *D. suzukii* is reported for the first time. Its role remains unclear based on the current literature. Although toluene is not produced directly by yeasts, it can result from the decomposition of terpenes [82]. It would be valuable to assess whether it is attractive to *D. suzukii.*

## Conclusion

We propose two effective yeast-based formulations for attract-and-kill strategies for *D. suzukii* that demonstrate no apparent impact on honeybee feeding behaviour. *Saccharomycopsis vini* is as effective as *H. uvarum* in enhancing the efficiency of an attract-and-kill strategy when combined with lower doses of spinosad compared to conventional methods, *S. vini* exhibits a different headspace profile compared to *H. uvarum* underscoring its potential as a new additional and different tool in integrated pest management. The reduced sugar syrup consumption by foraging bees further supports the compatibility of these strategies with honeybee safety. Additionally, the identification of 11 antennally active compounds provides a foundation for further research into the chemical cues driving yeast preference in *D. suzukii.* Future studies should explore the behavioural effects of these compounds on *D. suzukii* to optimise yeast-based pest management tools.

## Supporting information

S1 Fig Schematics of the arena trapping assay (A) and wind tunnel assay (B) to assess *Drosophila suzukii* behaviour. (PDF)

S2 Table Compounds identified in the headspaces of *Hanseniaspora uvarum, Saccharomycopsis vini* and *Saccharomyces cerevisiae* by solid phase microextraction followed by gas chromatograph-time of flight-mass spectrometry (SPME-GC-TOF-MS). (PDF)

S3 Table Summary of a chemical enrichment analysis (ChemRICH) for each class of the headspace compositions of (A) *Hanseniaspora uvarum* and *Saccharomycopsis vini*, (B) *H. uvarum* and *Saccharomyces cerevisiae* and C) *S. vini* and *S. cerevisiae.* (PDF)

S4 Table Electroantennography responses (EAG) of *Drosophila suzukii* to 40 compounds identified from headspaces of *Hanseniaspora uvarum, Saccharomycopsis vini* and *Saccharomyces cerevisiae.* (PDF)

S5 Fig Gas chromatography-electroantennography (GC-EAD) traces from antennae of *Drosophila suzukii* to 40 compounds identified from headspaces of *Hanseniaspora uvarum, Saccharomycopsis vini* and *Saccharomyces cerevisiae.* (PDF)

## References

[1] Walsh DB, Bolda MP, Goodhue RE, Dreves AJ, Lee J, Bruck DJ, et al. *Drosophila suzukii* (Diptera: Drosophilidae): invasive pest of ripening soft fruit expanding its geographic range and damage potential. J Integr Pest Manage. 2011;2(1):G1–7. doi: 10.1603/ipm10010

[2] Asplen MK, Anfora G, Biondi A, Choi D-S, Chu D, Daane KM, et al. Invasion biology of spotted wing Drosophila (*Drosophila suzukii*): a global perspective and future priorities. J Pest Sci. 2015;88(3):469–94. doi: 10.1007/s10340-015-0681-z

[3] Mori BA, Whitener AB, Leinweber Y, Revadi S, Beers EH, Witzgall P, et al. Enhanced yeast feeding following mating facilitates control of the invasive fruit pest *Drosophila suzukii*. J Appl Ecol. 2016;54(1):170–7. doi: 10.1111/1365-2664.12688

[4] Scheidler N, Liu C, Hamby K, Zalom F, Syed Z. Volatile codes: correlation of olfactory signals and reception in *Drosophila*-yeast chemical communication. Sci Rep. 2015;5(1):14059. 26391997 10.1038/srep14059 PMC4585764

[5] Chakraborty A, Mori B, Rehermann G, Hernández Garcia A, Lemmen‐Lechelt J, Hagman A, et al. Yeast and fruit fly mutual niche construction and antagonism against mould. Funct Ecol. 2022;36(7):1639–54. doi: 10.1111/1365-2435.14054

[6] Clyne P, Grant A, O’Connell R, Carlson JR. Odorant response of individual sensilla on the *Drosophila* antenna. Invert Neurosci. 1997;3(2–3):127–35. doi: 10.1007/BF02480367 9783438

[7] Hildebrand JG, Shepherd GM. Mechanisms of olfactory discrimination: converging evidence for common principles across phyla. Annu Rev Neurosci. 1997;20:595–631. doi: 10.1146/annurev.neuro.20.1.595 9056726

[8] Mombaerts P, Wang F, Dulac C, Chao S, Nemes A, Mendelsohn M, et al. Visualizing an olfactory sensory map. Cell. 1996;87(4):675–86. 8929536 10.1016/s0092-8674(00)81387-2

[9] Tait G, Mermer S, Stockton D, Lee J, Avosani S, Abrieux A, et al. *Drosophila suzukii* (Diptera: Drosophilidae): a decade of research towards a sustainable integrated pest management program. J Econ Entomol. 2021;114(5):1950–74. doi: 10.1093/jee/toab158 34516634

[10] Cini A, Ioriatti C, Anfora G. A review of the invasion of *Drosophila suzukii* in Europe and a draft research agenda for integrated pest management. Bull Insectol. 2012;65:149–60.

[11] Van Timmeren S, Isaacs R. Control of spotted wing drosophila, *Drosophila suzukii*, by specific insecticides and by conventional and organic crop protection programs. Crop Protect. 2013;54:126–33.

[12] Haviland DR, Beers EH. Chemical programs for *Drosophila suzukii* that comply with international limitations on pesticide residues for exported sweet cherries. J Integr Pest Manag. 2012;3(2):F1-6.

[13] Disi JO, Sial AA. Laboratory selection and assessment of resistance risk in *Drosophila suzukii* (Diptera: Drosophilidae) to Spinosad and Malathion. Insects. 2021;12(9):794. doi: 10.3390/insects12090794 34564234 PMC8466352

[14] Christen V, Krebs J, Bünter I, Fent K. Biopesticide spinosad induces transcriptional alterations in genes associated with energy production in honey bees (*Apis mellifera*) at sublethal concentrations. J Hazard Mater. 2019;378:120736. 31202068 10.1016/j.jhazmat.2019.06.013

[15] Mayes MA, Thompson GD, Husband B, Miles MM. Spinosad toxicity to pollinators and associated risk. Rev Environ Contam Toxicol. 2003;179:37–71. doi: 10.1007/0-387-21731-2_2 15366583

[16] Rhodes EM, Babu A, Sial AA, Liburd OE. Potential alternatives to spinosad as the killing agent mixed with two attractant products in attract-and-kill formulations used to manage the spotted-wing Drosophila, *Drosophila suzukii* (Diptera: Drosophilidae). J Econ Entomol. 2023;116(1):202–8. doi: 10.1093/jee/toac204 36617300

[17] Klick J, Rodriguez-Saona CR, Cumplido JH, Holdcraft RJ, Urrutia WH, da Silva RO, et al. Testing a novel attract-and-kill strategy for *Drosophila suzukii* (Diptera: Drosophilidae) Management. J Insect Sci. 2019;19(1):3. doi: 10.1093/jisesa/iey132 30624704 PMC6324652

[18] Ghosh S, Neupane R, Sahu DP, Teng J, Kong YL. The continuous actuation of liquid metal with a 3D-printed electrowetting device. Med X. 2025;3(1):9. doi: 10.1007/s44258-025-00052-8 40177535 PMC11958460

[19] Anton S, Cortesero A-M. Plasticity in chemical host plant recognition in herbivorous insects and its implication for pest control. Biology (Basel). 2022;11(12):1842. doi: 10.3390/biology11121842 36552352 PMC9775997

[20] Bellutti N, Gallmetzer A, Innerebner G, Schmidt S, Zelger R, Koschier EH. Dietary yeast affects preference and performance in *Drosophila suzukii*. J Pest Sci (2004). 2018;91(2):651–60. doi: 10.1007/s10340-017-0932-2 29568250 PMC5847167

[21] Hamby KA, Becher PG. Current knowledge of interactions between *Drosophila suzukii* and microbes, and their potential utility for pest management. J Pest Sci. 2016;89(3):621–30. doi: 10.1007/s10340-016-0768-1

[22] Knight AL, Basoalto E, Yee W, Hilton R, Kurtzman CP. Adding yeasts with sugar to increase the number of effective insecticide classes to manage *Drosophila suzukii* (Matsumura) (Diptera: Drosophilidae) in cherry. Pest Manag Sci. 2016;72(8):1482–90. doi: 10.1002/ps.4171 26454150

[23] Bianchi F, Spitaler U, Castellan I, Cossu CS, Brigadoi T, Duménil C, et al. Persistence of a yeast-based (*Hanseniaspora uvarum*) attract-and-kill formulation against *Drosophila suzukii* on grape leaves. Insects. 2020;11(11):810. doi: 10.3390/insects11110810 33217960 PMC7698740

[24] Spitaler U, Cossu CS, Delle Donne L, Bianchi F, Rehermann G, Eisenstecken D, et al. Field and greenhouse application of an attract-and-kill formulation based on the yeast *Hanseniaspora uvarum* and the insecticide spinosad to control *Drosophila suzukii* in grapes. Pest Manag Sci. 2022;78(3):1287–95. doi: 10.1002/ps.6748 34854220 PMC9299924

[25] Kleman I, Rehermann G, Kwadha CA, Witzgall P, Becher PG. *Hanseniaspora uvarum* attracts *Drosophila suzukii* (Diptera: Drosophilidae) with high specificity. J Econ Entomol. 2022;115(4):999–1007. doi: 10.1093/jee/toac029 35385117 PMC9365507

[26] Rehermann G, Spitaler U, Sahle K, Cossu CS, Donne LD, Bianchi F, et al. Behavioral manipulation of *Drosophila suzukii* for pest control: high attraction to yeast enhances insecticide efficacy when applied on leaves. Pest Manag Sci. 2022;78(3):896–904. doi: 10.1002/ps.6699 34716651

[27] Bjeljac M, Spitaler U, Mori N, Fusillo M, Bombardini E, Preti M, et al. Canopy strip applications of *Hanseniaspora uvarum* combined with spinosad reduce insecticide use without compromising *Drosophila suzukii* control in cherry. Crop Protection. 2024;186:106868. doi: 10.1016/j.cropro.2024.106868

[28] Spitaler U, Bianchi F, Eisenstecken D, Castellan I, Angeli S, Dordevic N, et al. Yeast species affects feeding and fitness of *Drosophila suzukii* adults. J Pest Sci. 2020;93(4):1295–309. doi: 10.1007/s10340-020-01266-y

[29] Castellan I, Duménil C, Rehermann G, Eisenstecken D, Bianchi F, Robatscher P, et al. Chemical and electrophysiological characterisation of headspace volatiles from yeasts attractive to *Drosophila suzukii*. J Chem Ecol. 2024;50(11):830–46. doi: 10.1007/s10886-024-01494-x 38691267 PMC11543737

[30] Anderson DL, Trueman JW. *Varroa jacobsoni* (Acari: Varroidae) is more than one species. Exp Appl Acarol. 2000;24(3):165–89. doi: 10.1023/a:1006456720416 11108385

[31] Ruebenbauer A, Schlyter F, Hansson BS, Löfstedt C, Larsson MC. Genetic variability and robustness of host odor preference in *Drosophila melanogaster*. Curr Biol. 2008;18(18):1438–43. doi: 10.1016/j.cub.2008.08.062 18804372

[32] Favaro R, Roved J, Haase A, Angeli S. Impact of chronic exposure to two neonicotinoids on honey bee antennal responses to flower volatiles and pheromonal compounds. Front Insect Sci. 2022;2:821145. doi: 10.3389/finsc.2022.821145 38468759 PMC10926470

[33] Alves Z, Melo A, Figueiredo AR, Coimbra MA, Gomes AC, Rocha SM. Exploring the *Saccharomyces cerevisiae* volatile metabolome: indigenous versus commercial strains. PLoS One. 2015;10(11):e0143641. doi: 10.1371/journal.pone.0143641 26600152 PMC4657929

[34] Tsugawa H, Cajka T, Kind T, Ma Y, Higgins B, Ikeda K, et al. MS-DIAL: data-independent MS/MS deconvolution for comprehensive metabolome analysis. Nat Methods. 2015;12(6):523–6. doi: 10.1038/nmeth.3393 25938372 PMC4449330

[35] Matheu MP, Cahalan MD, Parker I. General approach to adoptive transfer and cell labeling for immunoimaging. Cold Spring Harb Protoc. 2011;2011(2):pdb.prot5565. doi: 10.1101/pdb.prot5565 21285265

[36] Dobritsa AA, van der Goes van Naters W, Warr CG, Steinbrecht RA, Carlson JR. Integrating the molecular and cellular basis of odor coding in the *Drosophila antenna*. Neuron. 2003;37(5):827–41. 12628173 10.1016/s0896-6273(03)00094-1

[37] Hothorn T, Bretz F, Westfall P. Simultaneous inference in general parametric models. Biom J. 2008;50(3):346–63. doi: 10.1002/bimj.200810425 18481363

[38] Bates D, Mächler M, Bolker B, Walker S. Fitting linear mixed-effects models using lme4. J Stat Softw. 2015;67:1–48.

[39] Hartig F, Hartig M. Package ‘dharma’. https://cran.r-project.org/package=DHARMa.

[40] Graves S, Piepho H, Selzer L, DoraiRaj S. Multcompview: visualizations of paired comparisons. 2019.

[41] Weisberg S, Fox J. An R Companion to Applied Regression. 3rd ed. Sage Publications. 2019.

[42] Fan S, Kind T, Cajka T, Hazen SL, Tang WHW, Kaddurah-Daouk R. Systematic error removal using random forest for normalizing large-scale untargeted lipidomics data. Anal Chem. 2019;91(5):3590–6. 30758187 10.1021/acs.analchem.8b05592 PMC9652764

[43] Barupal DK, Fiehn O. Chemical similarity enrichment analysis (ChemRICH) as alternative to biochemical pathway mapping for metabolomic datasets. Sci Rep. 2017;7(1):14567. doi: 10.1038/s41598-017-15231-w 29109515 PMC5673929

[44] R core T. Stat package version 4.5.0. https://stat.ethz.ch/R-manual/R-devel/library/stats/html/00Index.html.

[45] Sharma A, Sandhi R, Reddy G. A review of interactions between insect biological control agents and semiochemicals. Insects. 2019;10(12):439. 31817457 10.3390/insects10120439 PMC6955951

[46] Gregg P, Socorro A, Landolt P. Advances in attract-and-kill for agricultural pests: beyond pheromones. Annu Rev Entomol. 2018;63. 10.1146/annurev-ento-031616-035040 29058978

[47] Bruce TJA, Pickett JA. Perception of plant volatile blends by herbivorous insects – finding the right mix. Phytochemistry. 2011 Sep 1;72(13):1605–11. 21596403 10.1016/j.phytochem.2011.04.011

[48] Jones R, Fountain MT, Günther CS, Eady PE, Goddard MR. Separate and combined *Hanseniaspora uvarum* and *Metschnikowia pulcherrima* metabolic volatiles are attractive to *Drosophila suzukii* in the laboratory and field. Sci Rep. 2021;11(1):1201. doi: 10.1038/s41598-020-79691-3 33441642 PMC7806593

[49] Erdei AL, Szelényi MO, Deutsch F, Rikk P, Molnár BP. Lure design for *Drosophila suzukii* based on liquid culture of fruit epiphytic yeasts: comparing the attractivity of fermentation volatiles for seasonal morphs. J Appl Entomol. 2022;146(6):773–85. doi: 10.1111/jen.13006

[50] Elsensohn JE, Burrack HJ. Plasticity in oviposition and foraging behavior in the invasive pest *Drosophila suzukii* across natural and agricultural landscapes. Ecol Evol. 2023;13(1):e9713. doi: 10.1002/ece3.9713 36620402 PMC9817201

[51] Cook DC, Thomas MB, Cunningham SA, Anderson DL, De Barro PJ. Predicting the economic impact of an invasive species on an ecosystem service. Ecol Appl. 2007;17(6):1832–40. doi: 10.1890/06-1632.1 17913144

[52] Egan PA, Dicks LV, Hokkanen HMT, Stenberg JA. Delivering integrated pest and pollinator management (IPPM). Trends Plant Sci. 2020;25(6):577–89. doi: 10.1016/j.tplants.2020.01.006 32407697

[53] Jacquet F, Jeuffroy M, Jouan J, Le Cadre E, Litrico I, Malausa T. Pesticide-free agriculture as a new paradigm for research. Agron Sustain Dev. 2022;42(1):8.

[54] Kevan P, Eisikowitch D, Fowle S, Thomas K. Yeast-contaminated nectar and its effects on bee foraging. J Apicult Res. 1988;27:26–9.

[55] Brysch-Herzberg M. Ecology of yeasts in plant-bumblebee mutualism in Central Europe. FEMS Microbiol Ecol. 2004;50(2):87–100. doi: 10.1016/j.femsec.2004.06.003 19712367

[56] Pozo MI, van Kemenade G, van Oystaeyen A, Aledón-Catalá T, Benavente A, Van den Ende W. The impact of yeast presence in nectar on bumble bee behavior and fitness. Ecol Monogr. 2020;90(1):e01393.

[57] Rering CC, Rudolph AB, Beck JJ. Pollen and yeast change nectar aroma and nutritional content alone and together, but honey bee foraging reflects only the avoidance of yeast. Environ Microbiol. 2021;23(8):4141–50. doi: 10.1111/1462-2920.15528 33876542

[58] Leach AB, Hoepting CA, Nault BA. Grower adoption of insecticide resistance management practices increase with extension-based program. Pest Manag Sci. 2019;75(2):515–26. doi: 10.1002/ps.5150 30047237

[59] Grabe V, Sachse S. Fundamental principles of the olfactory code. Biosystems. 2018;164:94–101. doi: 10.1016/j.biosystems.2017.10.010 29054468

[60] Duménil C, Woud D, Pinto F, Alkema JT, Jansen I, Van Der Geest AM, et al. Pheromonal cues deposited by mated females convey social information about egg-laying sites in *Drosophila melanogaster*. J Chem Ecol. 2016;42(3):259–69. doi: 10.1007/s10886-016-0681-3 26994611 PMC4839039

[61] Revadi S, Vitagliano S, Rossi Stacconi MV, Ramasamy S, Mansourian S, Carlin S, et al. Olfactory responses of *Drosophila suzukii* females to host plant volatiles. Physiol Entomol. 2015;40(1):54–64. doi: 10.1111/phen.12088

[62] Keesey IW, Knaden M, Hansson BS. Olfactory specialization in *Drosophila suzukii* supports an ecological shift in host preference from rotten to fresh fruit. J Chem Ecol. 2015;41(2):121–8. doi: 10.1007/s10886-015-0544-3 25618323 PMC4351439

[63] Feng Y, Bruton R, Park A, Zhang A. Identification of attractive blend for spotted wing drosophila, *Drosophila suzukii*, from apple juice. J Pest Sci (2004). 2018;91(4):1251–67. doi: 10.1007/s10340-018-1006-9 30100831 PMC6063330

[64] Cloonan KR, Abraham J, Angeli S, Syed Z, Rodriguez-Saona C. Advances in the chemical ecology of the spotted wing drosophila (*Drosophila suzukii*) and its applications. J Chem Ecol. 2018;44(10):922–39. 30054769 10.1007/s10886-018-1000-y

[65] Little CM, Dixon PL, Moreau DL, Chapman TW, Hillier NK. Assessment of attractant lures and monitoring traps for *Drosophila suzukii* (Diptera: Drosophidae) using electrophysiology, laboratory choice assays, and field trials. J Econ Entomol. 2021;114(2):652–75. doi: 10.1093/jee/toab006 33704447

[66] Wang Q, Xu P, Sanchez S, Duran P, Andreazza F, Isaacs R, et al. Behavioral and physiological responses of *Drosophila melanogaster* and *D. suzukii* to volatiles from plant essential oils. Pest Manag Sci. 2021;77(8):3698–705. doi: 10.1002/ps.6282 33442945

[67] Abraham J, Zhang A, Angeli S, Abubeker S, Michel C, Feng Y, et al. Behavioral and antennal responses of *Drosophila suzukii* (Diptera: Drosophilidae) to volatiles from fruit extracts. Environ Entomol. 2015;44(2):356–67. doi: 10.1093/ee/nvv013 26313190

[68] Kirkpatrick DM, Leach HL, Xu P, Dong K, Isaacs R, Gut LJ. Comparative antennal and behavioral responses of summer and winter morph *Drosophila suzukii* (Diptera: Drosophilidae) to ecologically relevant volatiles. Environ Entomol. 2018;47(3):700–6. doi: 10.1093/ee/nvy046 29668908

[69] Keesey IW, Jiang N, Weißflog J, Winz R, Svatoš A, Wang C-Z, et al. Plant-based natural product chemistry for integrated pest management of *Drosophila suzukii*. J Chem Ecol. 2019;45(7):626–37. doi: 10.1007/s10886-019-01085-1 31257561 PMC6661260

[70] Cha DH, Adams T, Rogg H, Landolt PJ. Identification and field evaluation of fermentation volatiles from wine and vinegar that mediate attraction of spotted wing *Drosophila*, *Drosophila* suzukii. J Chem Ecol. 2012;38(11):1419–31. doi: 10.1007/s10886-012-0196-5 23065086

[71] Renkema JM, Buitenhuis R, Hallett RH. Reduced *Drosophila suzukii* infestation in berries using deterrent compounds and laminate polymer flakes. Insects. 2017;8(4):117. doi: 10.3390/insects8040117 29088060 PMC5746800

[72] Aprea E, Biasioli F, Gasperi F. Volatile compounds of raspberry fruit: from analytical methods to biological role and sensory impact. Molecules. 2015;20(2):2445–74. doi: 10.3390/molecules20022445 25647579 PMC6272157

[73] Xinyu Z, Yi W, Xue H, Zixi Y, Weiqiong Y, Zhaolin L. Characterization of volatile compounds in five blueberry varieties using purge and trap coupled to gas chromatography-mass spectrometry. Ital J Food Sci. 2020;32(2).

[74] Cha DH, Adams T, Rogg H, Landolt PJ. Identification and field evaluation of fermentation volatiles from wine and vinegar that mediate attraction of spotted wing *Drosophila*, *Drosophila suzukii*. J Chem Ecol. 2012;38(11):1419–31. doi: 10.1007/s10886-012-0196-5 23065086

[75] Howell KS, Klein M, Swiegers JH, Hayasaka Y, Elsey GM, Fleet GH, et al. Genetic determinants of volatile-thiol release by *Saccharomyces cerevisiae* during wine fermentation. Appl Environ Microbiol. 2005;71(9):5420–6. doi: 10.1128/AEM.71.9.5420-5426.2005 16151133 PMC1214692

[76] Cornelius ML, Duan JJ, Messing RH. Volatile host fruit odors as attractants for the oriental fruit fly (Diptera: Tephritidae). J Econ Entomol. 2000;93(1):93–100. doi: 10.1603/0022-0493-93.1.93 14658517

[77] Tholl D, Boland W, Hansel A, Loreto F, Röse USR, Schnitzler J-P. Practical approaches to plant volatile analysis. Plant J. 2006;45(4):540–60. doi: 10.1111/j.1365-313X.2005.02612.x 16441348

[78] Carey A, Carlson J. Insect olfaction from model systems to disease control. Proc Natl Acad Sci U S A. 2011;108(32):12987–95. 21746926 10.1073/pnas.1103472108 PMC3156210

[79] Roelofs WL. Electroantennogram assays: rapid and convenient screening procedures for pheromones. In: Hummel HE, Miller TA, eds. Techniques in Pheromone Research. New York: Springer. 1984, pp. 131–59.

[80] Alcorta E. Characterization of the electroantennogram in *Drosophila melanogaster* and its use for identifying olfactory capture and transduction mutants. J Neurophysiol. 1991;65(3):702–14. doi: 10.1152/jn.1991.65.3.702 1904913

[81] Wallingford AK, Cha DH, Loeb GM. Evaluating a push-pull strategy for management of *Drosophila suzukii* Matsumura in red raspberry. Pest Manag Sci. 2018;74(1):120–5. doi: 10.1002/ps.4666 28714131

[82] Richards PI, Sandau CD. Forensic source attribution for toluene in environmental samples. Environ Toxicol Chem. 2018;37(3):729–37. doi: 10.1002/etc.4008 29044663

