# Supplementary material for: Yeast-based attract-and-kill strategies for Drosophila suzukii management without disrupting honey bee activity
Source: PLoS One. 2025 May 19;20(5):e0323653. doi: 10.1371/journal.pone.0323653 (PMC12088520; doi:10.1371/journal.pone.0323653)
Supplement: S1 Fig — (PDF) [file pone.0323653.s001.pdf]

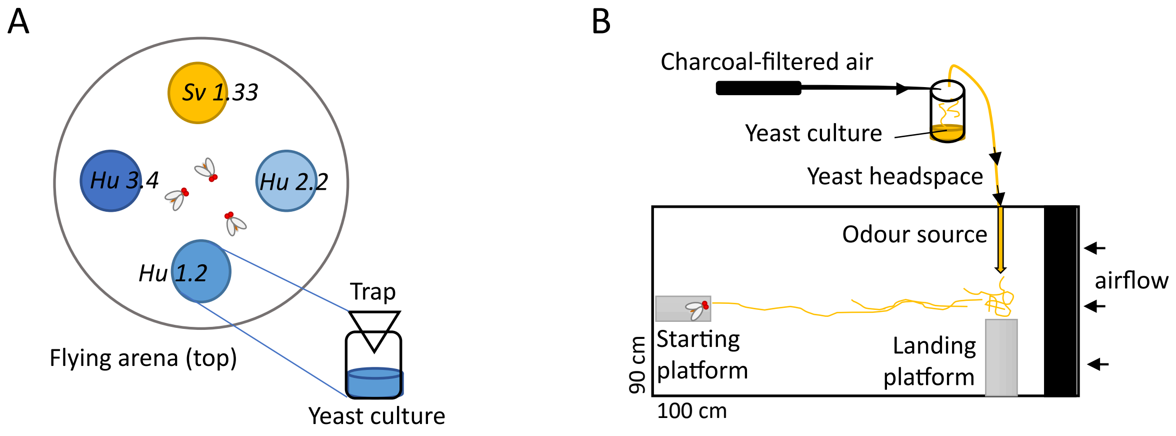

S1 Fig. Schematics of the arena trapping assay (A) and wind tunnel assay (B) to assess *Drosophila suzukii* behaviour toward volatiles of *Hanseniaspora uvarum* (strains Hu 1.21, Hu 2.2 and Hu 3.4), *Saccharomycopsis vini* (strain Sv 1.33) and *Saccharomyces cerevisiae* (strain S288c).
