# Supplementary material for: Yeast-based attract-and-kill strategies for Drosophila suzukii management without disrupting honey bee activity
Source: PLoS One. 2025 May 19;20(5):e0323653. doi: 10.1371/journal.pone.0323653 (PMC12088520; doi:10.1371/journal.pone.0323653)
Supplement: S2 Table — (PDF) [file pone.0323653.s002.pdf]

S2 Table. Compounds identified in the headspaces of *Hanseniaspora uvarum* strain 2.2 (Hu 2.2), *Saccharomycopsis vini* strain 1.33 (Sv 1.33) and *Saccharomyces cerevisiae* strain S288c (Sc S288c) by solid phase microextraction followed by gas chromatograph-time of flight-mass spectrometry (SPME-GC-TOF-MS).

Compound[1]	L.R.I [2]	mean ± SD peak height [3]			CAS number
		H.u. 2.2	S.v. 1.33	S.c. S288c	
**Alcohols and polyols**					
2-Methyl-1-propanol	1095	77160 ± 44022 [a]	64422 ± 26744 [a]	68698 ± 36671 [a]	78-83-1
1-Butanol	1145	1865 ± 745 [a]	2331 ± 1144 [a]	1651 ± 865 [a]	71-36-3
3-Methyl-1-butanol	1208	1140713 ± 307793 [a]	1434013 ± 225179 [a]	2309561 ± 836364 [a]	123-51-3
3-Methyl-3-buten-1-ol	1249	432 ± 282 [a]	807 ± 348 [a]	1290 ± 987 [a]	763-62-6
1-Pentanol	1249	609 ± 257 [a]	1642 ± 1151 [a]	626 ± 287 [a]	71-41-0
4-Penten-1-ol	1301	2005 ± 289 [a]	3525 ± 1303 [a]	2389 ± 431 [a]	821-09-0
4-Methyl -1-pentanol	1313	359 ± 37 [a]	2345 ± 681 [b]	395 ± 54 [a]	626-89-1
3-Methyl-1-pentanol	1325	590 ± 69 [a]	1077 ± 184 [b]	1703 ± 546 [c]	859-35-5
[R-(R*,R*)]-2,3-Butanediol	1543	171 ± 56 [a]	1391 ± 948 [b]	2132 ± 1061 [b]	24347-58-8
[S-(R*,R*)]-2,3-Butanediol	1581	826 ± 1212 [a]	392 ± 154 [a]	742 ± 353 [a]	19132-06-0
α-Cyclogeraniol	1688	87 ± 10 [a]	1238 ± 500 [b]	598 ± 509 [ab]	6627-74-3
1,4-Butanediol	1929	459 ± 174 [a]	425 ± 122 [a]	455 ± 113 [a]	110-63-4
**Aldehydes**					
3-methyl-Butanal	951	1202 ± 326 [a]	1204 ± 392 [a]	3289 ± 1012 [b]	590-86-3
5-Methyl-2-furaldehyde	1568	162 ± 59 [a]	214 ± 105 [a]	315 ± 277 [a]	620-02-0
Furfural	2274	612 ± 409 [a]	513 ± 100 [a]	1352 ± 1394 [a]	4602-84-0
5-Hydroxymethylfurfural	2378	146 ± 110 [a]	97 ± 15 [a]	535 ± 1070 [a]	67-47-0
**Alkanes**					
Ethyl heptanoate	1326	186 ± 34 [a]	494 ± 201 [b]	582 ± 230 [b]	106-30-9
Geranyl acetate	1754	514 ± 250 [a]	10973 ± 14694 [ab]	196 ± 69 [b]	105-87-3
**Benzene and substituted derivatives**					
Toluene	1231	565 ± 419 [a]	1534 ± 617 [b]	1420 ± 382 [b]	3779-61-1
p-(1-Propenyl)-toluene	1260	162 ± 51 [a]	3511 ± 1357 [b]	148 ± 42 [a]	99-87-6
p-Xylene	1270	438 ± 441 [a]	875 ± 549 [a]	879 ± 767 [a]	586-62-9
n-Butyl phthalate	1459	263 ± 59 [a]	293 ± 98 [a]	331 ± 81 [a]	1786-08-9
Benzaldehyde	1511	17065 ± 16355 [ab]	17484 ± 10837 [a]	5258 ± 1521 [b]	100-52-7
Diisobutyl phthalate	1634	657 ± 162 [a]	618 ± 149 [a]	913 ± 152 [b]	110-38-3
Phenylacetaldehyde	1634	6011 ± 6655 [a]	2788 ± 679 [ab]	10701 ± 5690 [b]	122-78-1
Ethyl benzoate	1656	201 ± 91 [a]	134 ± 44 [a]	140 ± 39 [a]	93-89-0
m-Cymene	1786	84 ± 37 [a]	5892 ± 6533 [a]	100 ± 59 [a]	535-77-3
Phenethyl acetate	1810	89529 ± 60120 [a]	5177 ± 4162 [b]	30176 ± 5580 [a]	103-45-7
Benzyl alcohol	1877	6191 ± 4583 [a]	4613 ± 2344 [a]	1269 ± 282 [b]	100-51-6
2-Phenylethanol	1910	582979 ± 130898 [a]	753122 ± 170800 [a]	1170504 ± 142925 [b]	60-12-8
Ethylbenzene	2217	122 ± 152 [a]	270 ± 296 [a]	289 ± 238 [a]	54546-22-4
2,4-Di-tert-butylphenol	2253	1689 ± 196 [a]	1996 ± 681 [ab]	2925 ± 1004 [b]	96-76-4
Methyl N-hydroxybenzenecarboximido	2499	2593 ± 2871 [a]	1828 ± 1688 [a]	2372 ± 2089 [a]	84-74-2
2,6-Di-tert-butylhydroquinone	2616	859 ± 54 [a]	1320 ± 223 [b]	1061 ± 534 [ab]	2444-28-2
**Branched fatty acids**					
2-Methylbutanoic acid	1707	7546 ± 5340 [a]	12013 ± 3642 [a]	30436 ± 4740 [b]	116-53-0
3-Methylbutanoic acid	1708	3740 ± 1041 [a]	4068 ± 1335 [a]	16024 ± 3684 [b]	503-74-2
**Branched unsaturated hydrocarbons**					
3,3,5-Trimethyl-1,5-heptadiene	1099	116 ± 29 [a]	2159 ± 2305 [a]	108 ± 39 [a]	74630-29-8
3,4-Dimethyl-2,4,6-octatriene	1365	353 ± 51 [a]	86059 ± 38464 [b]	352 ± 120 [a]	57396-75-5
γ-Terpinene	1787	85 ± 20 [a]	2047 ± 1154 [b]	70 ± 17 [a]	13066-51-8
**Carboxylic acid esters**					
Ethyl acetate	941	1747424 ± 265873 [a]	1217426 ± 598476 [a]	33251 ± 2045 [b]	141-78-6
Pentyl 2-methylpropanoate	972	200 ± 27 [a]	733 ± 311 [b]	541 ± 164 [b]	105-37-3
Propyl acetate	984	7154 ± 3086 [a]	6109 ± 3540 [a]	804 ± 137 [b]	109-60-4
Isobutyl acetate	1011	15486 ± 3650 [a]	947 ± 233 [b]	2105 ± 857 [c]	110-19-0
n-Butyl acetate	1068	1905 ± 176 [a]	1406 ± 480 [ab]	1976 ± 318 [b]	123-86-4
2-methylbutyl acetate	1115	295741 ± 101588 [a]	11027 ± 2266 [b]	60878 ± 7238 [c]	624-41-9
Isoamyl propanoate	1123	2133 ± 1991 [a]	4310 ± 4145 [a]	1082 ± 211 [a]	106-42-3
Isoamyl acetate	1127	22368 ± 31346 [a]	1096 ± 539 [a]	3234 ± 2623 [a]	123-92-2

Ethyl propanoate	1181	3330 ± 1690 [a]	12562 ± 3900 [b]	1020 ± 98 [c]	105-68-0
3-Methyl-3-butenyl acetate	1193	363 ± 110 [ab]	265 ± 255 [a]	93 ± 15 [b]	1207311
3-(Methylthio)propyl acetate	1623	1171 ± 556 [a]	285 ± 149 [b]	228 ± 137 [b]	16630-55-0
**Carboxylic acids**					
2-methylpropanoic acid	1256	15788 ± 6187 [a]	9916 ± 3108 [a]	30643 ± 3762 [b]	2445-72-9
Acetic acid	1493	21260 ± 3895 [a]	11189 ± 10038 [a]	17617 ± 2854 [a]	64-19-7
Propanoic acid	1606	188 ± 179 [a]	106 ± 88 [a]	309 ± 392 [a]	79-31-2
**Dialkylthioethers**					
3-(methylthio)-1-Propanol	1715	4057 ± 713 [a]	17031 ± 1916 [b]	6853 ± 563 [c]	505-10-2
**Dicarboxylic acids and derivatives**					
Texanol Isobutanoate	1871	1110 ± 167 [a]	712 ± 414 [a]	1325 ± 639 [a]	6846-50-0
**Ethers**					
3-Ethoxy-1-propanol	1370	1055 ± 897 [a]	950 ± 463 [a]	910 ± 132 [a]	111-35-3
**Fatty acid esters**					
Ethyl 9-decenoate	961	127 ± 34 [a]	222 ± 179 [a]	8419 ± 3224 [b]	64-17-5
Ethyl butanoate	1033	360 ± 73 [a]	1528 ± 598 [b]	1358 ± 299 [b]	105-54-4
Ethyl hexanoate	1224	1076 ± 350 [a]	2322 ± 1269 [a]	37933 ± 13445 [b]	123-66-0
Ethyl tetradecanoate	1242	789 ± 171 [a]	1062 ± 461 [a]	676 ± 164 [a]	288-47-1
Hexyl 2-methylbutanoate	1420	247 ± 157 [a]	162 ± 69 [a]	119 ± 76 [a]	10032-15-2
3-methylbutyl octanoate	1427	314 ± 211 [a]	214 ± 142 [a]	2555 ± 808 [b]	106-32-1
Ethyl octanoate	1427	7205 ± 2609 [a]	2678 ± 968 [b]	245010 ± 64693 [c]	539-12-8
Isopentyl hexanoate	1453	124 ± 29 [a]	454 ± 174 [b]	458 ± 274 [b]	2198-61-0
3-Methylbutyl nonanoate	1531	637 ± 262 [a]	779 ± 83 [a]	788 ± 169 [a]	123-29-5
Isoamyl decanoate	1583	183 ± 73 [a]	896 ± 558 [b]	10636 ± 4652 [c]	79-09-4
Hexadecane	1597	180 ± 42 [a]	405 ± 203 [b]	308 ± 113 [b]	544-76-3
Hexyl hexanoate	1604	153 ± 64 [a]	127 ± 15 [a]	120 ± 54 [a]	6378-65-0
Phenethyl octanoate	1653	72 ± 20 [a]	96 ± 22 [a]	1045 ± 370 [b]	2035-99-6
Ethyl 9-hexadecenoate	1686	3966 ± 2261 [a]	4365 ± 2354 [a]	1215 ± 135 [b]	67233-91-4
Heptadecane	1697	199 ± 135 [a]	148 ± 71 [a]	287 ± 201 [a]	629-78-7
Phenethyl decanoate	1752	83 ± 15 [a]	116 ± 81 [a]	4586 ± 2231 [b]	30673-38-2
Isobutyl nonanoate	1756	477 ± 98 [a]	565 ± 154 [ab]	716 ± 171 [b]	7779-70-6
Hexanoic acid	1885	1919 ± 343 [a]	2490 ± 740 [a]	46736 ± 14570 [b]	142-62-1
Ethyl dodecanoate	2053	1038 ± 184 [a]	452 ± 220 [b]	9199 ± 3351 [c]	6309-51-9
Ethyl nonanoate	2079	1373 ± 666 [a]	2727 ± 914 [b]	3341 ± 985 [b]	124-07-2
2-Phenylethyl hexanoate	2130	83 ± 10 [a]	121 ± 51 [a]	1677 ± 654 [b]	6290-37-5
isobutyl decanoate	2243	89 ± 10 [a]	87 ± 32 [a]	880 ± 228 [b]	334-48-5
Isobutyl octanoate	2286	109 ± 56 [a]	46 ± 10 [b]	555 ± 110 [c]	5457-70-5
isobutyl decanoate	2393	75 ± 10 [a]	93 ± 12 [b]	1205 ± 441 [c]	143-07-7
Ethyl decanoate	2434	3193 ± 916 [a]	3052 ± 2761 [a]	91486 ± 29712 [b]	61810-55-7
Isoamyl laurate	2570	100 ± 34 [a]	246 ± 171 [a]	5597 ± 2300 [b]	6309-54-2
**Fatty alcohols**					
1-Hexanol	1352	1283 ± 287 [ab]	1585 ± 110 [a]	1218 ± 189 [b]	111-27-3
1-Octen-3-ol	1450	133 ± 125 [a]	827 ± 519 [b]	81 ± 22 [a]	3391-86-4
1-Heptanol	1454	11808 ± 4874 [a]	2545 ± 522 [b]	126349 ± 32069 [c]	111-70-6
2-Ethyl-1-hexanol	1489	8955 ± 1526 [a]	6833 ± 1852 [ab]	6177 ± 1271 [b]	104-76-7
2-Nonanol	1519	3160 ± 265 [a]	634 ± 179 [b]	406 ± 110 [c]	628-99-9
1-Octanol	1557	1242 ± 247 [a]	1355 ± 198 [a]	1433 ± 299 [a]	111-87-5
1-Nonanol	1660	1567 ± 235 [a]	681 ± 73 [b]	1605 ± 360 [a]	143-08-8
α-Citronellol	1760	82 ± 29 [a]	2321 ± 1043 [b]	91 ± 20 [a]	141-25-3
1-Decanol	1763	1159 ± 149 [a]	845 ± 81 [b]	1185 ± 318 [a]	112-30-1
**Fatty alcohol esters**					
Undecyl butanoate	1867	313 ± 76 [a]	271 ± 81 [a]	292 ± 29 [a]	´5461-02-9
**Heteroaromatic compounds**					
Furfuryl acetate	1538	605 ± 321 [a]	77 ± 27 [b]	126 ± 29 [c]	623-17-6
2-Furanmethanol	1666	1696 ± 240 [a]	1284 ± 96 [b]	1664 ± 816 [ab]	98-00-0
**Hydroxy fatty acids**					
4-Hydroxybutanoate	1616	515 ± 230 [a]	632 ± 277 [a]	506 ± 282 [a]	591-81-1
**Indoles**					
2-(1H-Indol-3-yl)ethyl acetate	2907	1014 ± 919 [a]	91 ± 10 [a]	98 ± 20 [a]	13137-14-9

**Ketones**					
Acetone	923	2936 ± 541 [a]	4671 ± 1217 [b]	4760 ± 517 [b]	67-64-1
2,3-Pentanedione	1061	1220 ± 625 [a]	651 ± 103 [a]	555 ± 220 [a]	600-14-6
2-Heptanone	1173	3298 ± 1070 [a]	6878 ± 4171 [a]	4321 ± 1095 [a]	110-43-0
Acetoin	1280	42568 ± 12698 [a]	2059 ± 2082 [b]	10715 ± 3780 [c]	513-86-0
Acetol	1295	862 ± 620 [a]	1007 ± 257 [a]	3493 ± 2655 [a]	116-09-6
2-Nonanone	1378	4038 ± 1531 [a]	12688 ± 4247 [b]	2848 ± 813 [a]	821-55-6
2-Methylthiolan-3-one	1516	896 ± 78 [a]	221 ± 83 [b]	1557 ± 252 [c]	13679-85-1
2-Undecanone	1590	277 ± 37 [a]	2470 ± 534 [b]	591 ± 137 [c]	112-12-9
Acetophenone	1638	583 ± 39 [a]	656 ± 22 [b]	661 ± 71 [b]	98-86-2
2-Acetylthiazole	1639	1558 ± 105 [a]	1591 ± 125 [a]	691 ± 174 [b]	24295-03-2
2-Hydroxy-iso-butyrophenone	2063	432 ± 257 [a]	923 ± 534 [a]	433 ± 203 [a]	7473-98-5
**Lactones**					
2-Hydroxy-gamma-butyrolactone	2139	276 ± 61 [a]	206 ± 44 [a]	515 ± 558 [a]	19444-84-9
4-Hydroxydihydro-2(3H)-furanone	2444	204 ± 49 [a]	182 ± 24 [a]	365 ± 468 [a]	5469-16-9
**Medium-chain fatty acids**					
Decanoic acid	1179	876 ± 287 [a]	574 ± 644 [a]	42893 ± 12267 [b]	5989-27-5
Dodecanoic acid	1360	109 ± 37 [a]	105 ± 34 [a]	1393 ± 897 [b]	3658-80-8
Octanoic acid	1547	6972 ± 1646 [a]	1383 ± 1384 [b]	100883 ± 32044 [c]	5461-06-3
Nonanoic acid	1650	1264 ± 465 [a]	579 ± 269 [b]	2795 ± 1017 [c]	30982-03-7
Ethyl hexadecanoate	2199	1703 ± 919 [ab]	2363 ± 921 [a]	783 ± 191 [b]	628-97-7
9-Decenoic acid	2290	93 ± 29 [a]	94 ± 10 [a]	1156 ± 593 [b]	14436-32-9
**Monoterpenoids**					
(+)-Sabinene	1145	104 ± 73 [a]	7567 ± 5960 [b]	142 ± 181 [a]	2009-00-9
β-Myrcene	1153	201 ± 333 [a]	176450 ± 83831 [b]	133 ± 105 [a]	123-35-3
(+)-4-Carene	1160	75 ± 34 [a]	3201 ± 3268 [a]	75 ± 20 [a]	29050-33-7
β-Phellandrene	1187	87 ± 24 [a]	11165 ± 5622 [b]	110 ± 34 [a]	555-10-2
(+)-Sylvestrene	1190	122 ± 93 [a]	5021 ± 5431 [a]	107 ± 44 [a]	1461-27-4
(Z)-β-Ocimene	1244	150 ± 135 [a]	114930 ± 58883 [b]	85 ± 49 [a]	3338-55-4
Linalool	1254	1189 ± 426 [a]	195699 ± 86629 [b]	590 ± 323 [c]	535-77-3
Geranial	1261	523 ± 135 [a]	46115 ± 18435 [b]	341 ± 115 [c]	99-85-4
Isoterpinolene	1266	83 ± 15 [a]	6391 ± 2976 [b]	76 ± 27 [a]	586-63-0
Isobutyl hexanoate	1348	136 ± 12 [a]	192 ± 37 [b]	367 ± 118 [c]	105-79-3
(4E,6Z)-Allocimene	1363	93 ± 15 [a]	13427 ± 16627 [a]	82 ± 32 [a]	7216-56-0
(4E,6E)-Allocimene	1383	78 ± 29 [a]	3069 ± 4005 [a]	44 ± 29 [a]	3016-19-1
(4Z,6Z)-Allocimene	1385	94 ± 42 [a]	20701 ± 21759 [b]	86 ± 34 [a]	17202-20-9
γ-Isogeraniol	1462	937 ± 164 [a]	11388 ± 5036 [b]	2685 ± 622 [c]	98-01-1
Limonene	1549	97 ± 66 [a]	23163 ± 11488 [b]	129 ± 88 [a]	78-70-6
α-Terpineol	1691	237 ± 56 [a]	12503 ± 5776 [b]	407 ± 91 [c]	98-55-5
(E)-β-Ocimene	1724	115 ± 93 [a]	76852 ± 37254 [b]	81 ± 27 [a]	5392-40-5
β-Citronellol	1768	1272 ± 534 [a]	178389 ± 80138 [b]	1110 ± 299 [a]	106-22-9
(Z)-Geraniol	1800	922 ± 429 [a]	126173 ± 63976 [b]	2476 ± 566 [c]	106-25-2
(Z)-Isogeraniol	1815	293 ± 44 [a]	1575 ± 554 [b]	717 ± 174 [c]	5944-20-7
Nerol	1850	7863 ± 1791 [a]	1669510 ± 511392 [b]	3669 ± 870 [c]	106-24-1
p-Cymene	1860	163 ± 76 [a]	8290 ± 4034 [b]	89 ± 32 [c]	2306-91-4
Terpinolene	2039	97 ± 32 [ab]	2250 ± 2778 [a]	83 ± 20 [b]	124-06-1
**Olefins**					
α-Ocimene	1246	184 ± 64 [a]	37933 ± 44439 [a]	123 ± 51 [a]	1002-35-3
(3E,5E)-2,6-Dimethyl-1,3,5,7-octatetra	1439	116 ± 22 [a]	6447 ± 2822 [b]	86 ± 17 [c]	460-01-5
**Organic trisulfides**					
Dimethyl trisulfide	2398	79 ± 10 [a]	88 ± 15 [a]	73 ± 7 [a]	84-69-5
**Oxanes**					
(Z)-Rose oxide	1341	68 ± 10 [a]	1821 ± 1291 [b]	81 ± 17 [a]	16409-43-1
Tetrahydro-2,5-dimethyl-2H-pyranmetl	1400	275 ± 277 [a]	337 ± 502 [a]	152 ± 54 [a]	54004-46-5
**Primary alcohols**					
Ethanol	1841	672262 ± 96243 [a]	664801 ± 117754 [a]	949533 ± 60414 [b]	106-33-2
**Pyrans**					
Nerol oxide	2166	71 ± 17 [a]	1732 ± 1337 [b]	59 ± 12 [a]	112-05-0
**Pyrazines**					

2,6-Dimethylpyrazine	1308	529 ± 103 [a]	558 ± 69 [a]	574 ± 225 [a]	108-50-9
Trimethylpyrazine	1388	436 ± 47 [a]	695 ± 137 [b]	582 ± 93 [b]	14667-55-1
**Sesquiterpenoids**					
Farnesol 3	1111	4906 ± 2060 [a]	5197 ± 2214 [a]	2943 ± 845 [a]	100-41-4
(Z)-β-Farnesene	1661	6730 ± 1656 [a]	5094 ± 1151 [a]	3786 ± 1404 [b]	18794-84-8
(E,Z)-α-Farnesene	1722	817 ± 108 [a]	691 ± 701 [ab]	435 ± 213 [b]	28973-98-0
(E,E)-α-Farnesene	1745	1998 ± 532 [a]	1654 ± 291 [ab]	1139 ± 576 [b]	502-61-4
(6E)-Nerolidol	2037	1809 ± 794 [a]	836 ± 169 [b]	1512 ± 884 [ab]	1119-38-6
2,3-Dihydrofarnesol	2211	1283 ± 83 [a]	7670 ± 2334 [b]	1579 ± 465 [a]	27745-36-4
(2Z,6E)-Farnesol	2244	80 ± 22 [a]	98 ± 32 [a]	332 ± 108 [b]	3790-71-4
**Straight chain fatty acids**					
Butanoic acid	1669	12722 ± 2966 [a]	28076 ± 4958 [b]	18292 ± 5729 [a]	107-92-6
**Thiazoles**					
Thiazole	1031	2733 ± 524 [a]	3021 ± 762 [a]	3603 ± 794 [a]	108-88-3

[1]Headspace compounds were identified and annotated following a collection by solid-phase microextraction and gas chromatography-time of flight-mass spectrometry (SPME-GC-TOF-MS). [2] Linear Retention Index on ZB-WAX column.[3]Mean ± standard deviation (SD) peak height. Numbers with different letters are significantly different (P<0.05) following a a Kolmogorov–Smirnov-test through a chemical enrichment (ChemRICH) analysis .
