# Supplementary material for: Yeast-based attract-and-kill strategies for Drosophila suzukii management without disrupting honey bee activity
Source: PLoS One. 2025 May 19;20(5):e0323653. doi: 10.1371/journal.pone.0323653 (PMC12088520; doi:10.1371/journal.pone.0323653)
Supplement: S3 Table — (PDF) [file pone.0323653.s003.pdf]

S3 Table. Results of a chemical enrichment analysis (ChemRICH) for each class of the headspace compositions of A) *Hanseniaspora uvarum* strain 2.2 and *Saccharomycopsis vini* strain 1.33, B) *H. uvarum* and *Saccharomyces cerevisiae* strain S288c, and C) *S. vini* and *S. cerevicae* .

Class[1]	Cluster size	*P* -values[2]	FDR-adjusted *P* -value[3]	Altered metabolites	Increased	Decreased	Increased ratio	Altered Ratio
A H. uvarum* vs *S. vini								
Alcohols and polyols	12	1.4E-04	3.3E-04	5	0	5	0	0.4
Benzene and substituted derivatives	16	0.012	0.018	4	1	3	0.2	0.2
Branched unsaturated hydrocarbons	3	5.5E-04	9.4E-04	2	0	2	0	0.7
Carboxylic acid esters	11	3.3E-04	6.6E-04	5	3	2	0.6	0.5
Fatty acid esters	26	6.6E-07	2.6E-06	10	3	7	0.3	0.4
Fatty alcohols	9	1.1E-07	6.3E-07	7	4	3	0.6	0.8
Ketones	9	4.7E-05	1.4E-04	4	0	4	0	0.4
Medium-chain fatty acids	6	0.15	0.18	2	2	0	1	0.3
Monoterpenoids	22	2.2E-20	2.6E-19	17	0	17	0	0.8
Sesquiterpenoids	7	0.065	0.086	2	1	1	0.5	0.3
B H. uvarum* vs *S. cerevicae								
Alcohols and polyols	12	0.016	0.024	3	0	3	0	0.2
Benzene and substituted derivatives	16	2.0E-04	4.7E-04	5	1	4	0.2	0.3
Branched unsaturated hydrocarbons	3	1	1	0	0	0		0
Carboxylic acid esters	11	2.3E-06	9.4E-06	8	7	1	0.9	0.7
Fatty acid esters	26	2.2E-20	2.6E-19	21	1	20	0.05	0.8
Fatty alcohols	9	0.12	0.16	3	2	1	0.7	0.3
Ketones	9	3.8E-06	1.1E-05	5	2	3	0.4	0.6
Medium-chain fatty acids	6	2.5E-11	1.5E-10	6	0	6	0	1
Monoterpenoids	22	0.0049	8.5E-03	7	3	4	0.4	0.3
Sesquiterpenoids	7	5.8E-04	1.2E-03	4	3	1	0.8	0.6
C S. vini vs S. cerevicae								
Alcohols and polyols	12	5.2E-04	7.1E-04	2	1	1	0.5	0.2
Benzene and substituted derivatives	16	4.9E-04	7.1E-04	7	4	3	0.6	0.4
Branched unsaturated hydrocarbons	3	5.3E-04	7.1E-04	2	0	2	0	0.7
Carboxylic acid esters	11	4.8E-06	1.5E-05	6	3	3	0.5	0.5
Fatty acid esters	26	6.9E-11	2.7E-10	17	15	2	0.9	0.7
Fatty alcohols	9	9.8E-06	2.4E-05	7	3	4	0.4	0.8
Ketones	9	0.01	0.012	3	0	3	0	0.3
Medium-chain fatty acids	6	2.3E-11	1.4E-10	6	6	0	1	1
Monoterpenoids	22	2.2E-20	2.6E-19	17	0	17	0	0.8
Sesquiterpenoids	7	1.1E-04	2.1E-04	2	1	1	0.5	0.3

[1]Chemical classes from headspaces identified and annotated following a collection by solid-phase microextraction and gas chromatography-time of flight-mass spectrometry (SPME-GC-TOF-MS). [2]Following a Kolmogorov–Smirnov-test. [3]Following a false discovery rate (FDR) correction
