# Supplementary material for: Yeast-based attract-and-kill strategies for Drosophila suzukii management without disrupting honey bee activity
Source: PLoS One. 2025 May 19;20(5):e0323653. doi: 10.1371/journal.pone.0323653 (PMC12088520; doi:10.1371/journal.pone.0323653)
Supplement: S4 Table — (PDF) [file pone.0323653.s004.pdf]

S4 Table. Electroantennography responses (EAG) of *Drosophila suzukii* to 40 compounds identified from headspaces of *Hanseniaspora uvarum, Saccharomycopsis vini* and *Saccharomyces cerevisiae*

Compound[1]	Amplitude (mV)[2]	Statistics[3]
**Alcohols and polyols**		
3-Methyl-1-butanol	-24.12 ± 22.68	t_8 = 2.39; *P* = 0.044
(R,R)-2,3-Butanediol	-29.67 ± 30.66	t_8 = 2.03; *P* = 0.077
4-Methyl-1-pentanol	-29.67 ± 22.85	t_5 = 5.12; *P* = 0.004
3-Methyl-1-pentanol	-11.94 ± 6.28	t_5 = 2.34; *P* = 0.067
**Benzene and substituted derivatives**		
Toluene	-15.13 ± 15.02	t_8 = 1.25; *P* = 0.248
2-Phenylethanol	-6.62 ± 7.18	t_8 = 1.95; *P* = 0.087
2-Phenylethyl acetate	-39.95 ± 30.34	t_5 = 2.21; *P* = 0.078
**Branched unsaturated hydrocarbons**		
gamma-Terpinene	0.12 ± 0.14	t_3 = -14.15; *P* = 0.001
**Carboxylic acid esters**		
Ethyl acetate	-60.46 ± 26.21	t_5 = 4.01; *P* = 0.01
Ethyl propanoate	-36.7 ± 42.23	t_8 = 2.29; *P* = 0.041
Isobutyl acetate	-30.1 ± 19.57	t_5 = 7.88; *P* = 0.001
2-Methylbutyl acetate	-27.26 ± 15.6	t_5 = 6.33; *P* = 0.001
**Fatty acid esters**		
Ethyl butanoate	-50.14 ± 16.66	t_5 = 3.04; *P* = 0.029
Ethyl octanoate	0.1 ± 0.15	t_5 = -2.85; *P* = 0.036
**Fatty alcohol**		
1-Heptanol	-13.63 ± 14.99	t_5 = 2.41; *P* = 0.061
2-Nonanol	-8.61 ± 11.68	t_5 = 1.26; *P* = 0.262
**Ketones**		
Acetone	-20.02 ± 22.29	t_8 = 1.82; *P* = 0.106
Acetoin	-51.09 ± 48.21	t_8 = 2.32; *P* = 0.049
2-Methylthiolan-3-one	-13.83 ± 13.29	t_8 = 2.45; *P* = 0.04
2-Acetylthiazole	-5.08 ± 9.28	t_5 = 0.19; *P* = 0.855
2-Undecanone	1.14 ± 9.82	t_5 = -0.06; *P* = 0.954
**Medium-chain fatty acids**		
Hexanoic acid	-13.94 ± 16.65	t_8 = 0.97; *P* = 0.363
Octanoic acid	-9.19 ± 27.81	t_8 = -1.41; *P* = 0.197
Nonanoic acid	3.95 ± 6.12	t_8 = -3.96; *P* = 0.004
Decanoic acid	-10.14 ± 16.53	t_8 = -0.34; *P* = 0.743
**Monoterpenoids**		
β-Myrcene	-0.48 ± 0.87	t_8 = -0.52; *P* = 0.618
Limonene	-7.76 ± 14.31	t_9 = -0.59; *P* = 0.572
p-Cymene	-17.33 ± 30.6	t_9 = 0.81; *P* = 0.436
(Z)-β-Ocimene[+]	-0.51 ± 0.36	t_9 = -1.55; *P* = 0.157
(E)-β-Ocimene[+]	-0.51 ± 0.36	t_9 = -1.55; *P* = 0.157
Linalool	-1.63 ± 1.32	t_8 = 0.42; *P* = 0.689
(4Z,6Z)-Allocimene[+]	-0.88 ± 0.96	t_9 = 2.38; *P* = 0.041
alpha-Terpineol	-5.2 ± 12.15	t_9 = -1.4; *P* = 0.195
(Z)-Geraniol	-3.18 ± 5.49	t_9 = -3.23; *P* = 0.01
Citronellol	-11.74 ± 15.15	t_9 = 0.99; *P* = 0.348
Nerol	-10.23 ± 12.48	t_9 = 1.23; *P* = 0.251
Geranial	-9.51 ± 13.89	t_9 = -0.99; *P* = 0.347
**Sesquiterpenoids**		
(E,Z)-alpha-Farnesene[+]	-2.73 ± 4.29	t_5 = 0.52; *P* = 0.628
(6E)-Nerolidol	-7.15 ± 13.55	t_5 = 1.41; *P* = 0.216
(2Z,6E)-Farnesol[+]	-5.26 ± 3.85	t_5 = 1.02; *P* = 0.356

[1] Compounds identified as significantly different between the three yeasts species by solid phase microextraction followed by gas chromatograph-time of flight-mass spectrometry (SPME-GC-TOF-MS) and chemical similarity enrichment analysis (ChemRICH). [+] The standard solution included several isomers. [2] Mean ± SD amplitude (mV) of antennal response in electroantennography (EAG) recordings (n = 6-10). [3] Two tailed paired t-test between standard and paraffin oil (control). Standards were of dilution 10^{-3} v/v.
