# Supplementary material for: Yeast-based attract-and-kill strategies for Drosophila suzukii management without disrupting honey bee activity
Source: PLoS One. 2025 May 19;20(5):e0323653. doi: 10.1371/journal.pone.0323653 (PMC12088520; doi:10.1371/journal.pone.0323653)
Supplement: S5 Fig — (PDF) [file pone.0323653.s005.pdf]

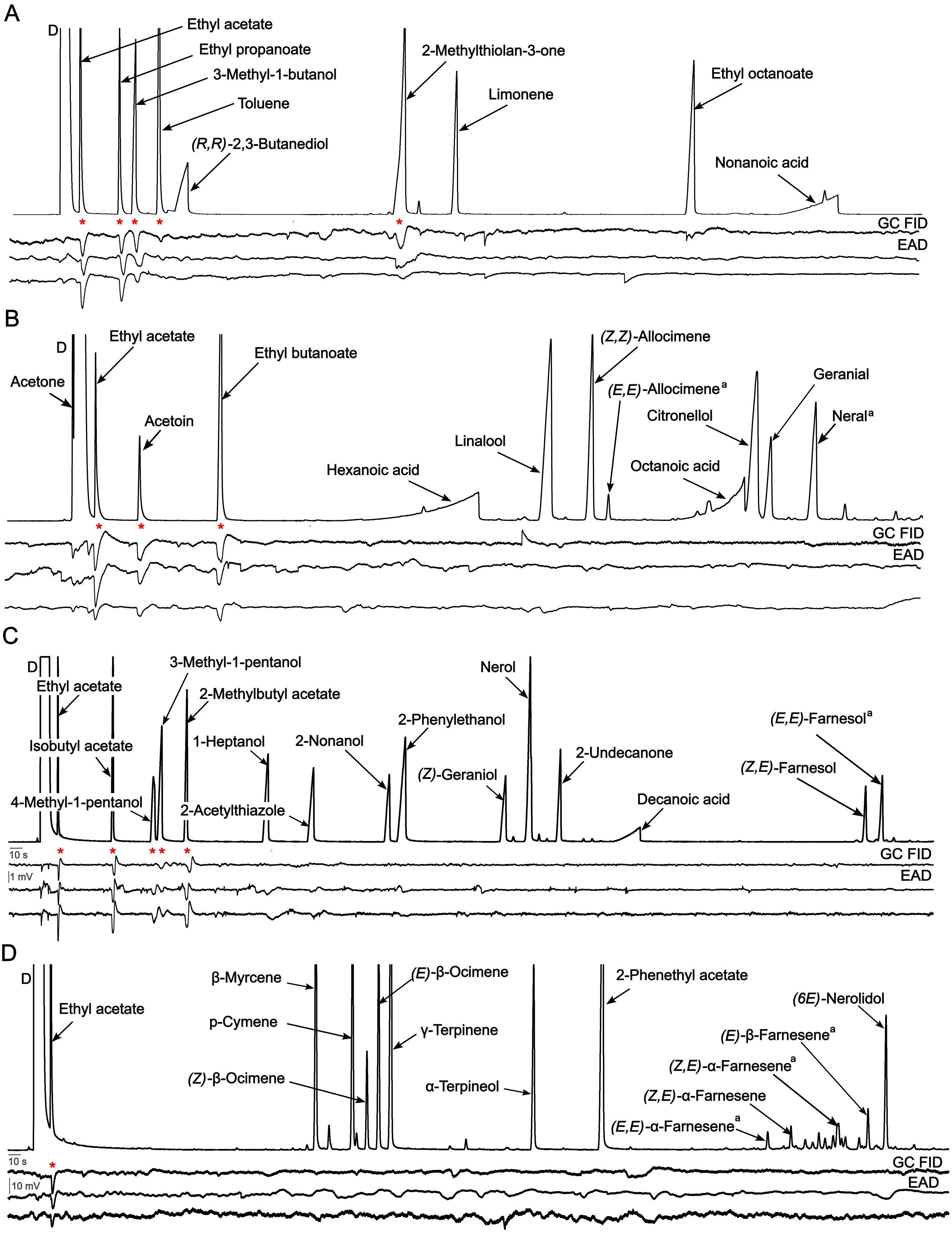

S5 Fig. Gas chromatography-electroantennography (GC-EAD) traces from antenna of *Drosophila suzukii* to 40 compounds of *Hanseniaspora uvarum, Saccharomycopsis vini,* and *Saccharomyces cerevisiae.*

Standards were eluted in isomix (a-d) with a 10^{-3} v/v dilution in dichloromethane (D) through a flame ionizing detector (FID) and simultaneously on the antenna (EAD). Ethyl acetate was included in each mix as a positive control. The EAD traces of three females are shown as example. *Responses in 100% of the 6-10 flies are indicated with red asterisks. 6-10 flies were tested, and no differences were observed between males and females.[a] Other chemicals present in isomeric standard mixtures.
